# The Gut Microbiota Determines the High-Altitude Adaptability of Tibetan Wild Asses (*Equus kiang*) in Qinghai-Tibet Plateau

**DOI:** 10.3389/fmicb.2022.949002

**Published:** 2022-07-18

**Authors:** Hongjin Liu, Xueping Han, Na Zhao, Linyong Hu, Xungang Wang, Chongliang Luo, Yongwei Chen, Xinquan Zhao, Shixiao Xu

**Affiliations:** ^1^Northwest Institute of Plateau Biology and Institute of Sanjiangyuan National Park, Chinese Academy of Sciences, Xining, China; ^2^Key Laboratory of Adaptation and Evolution of Plateau Biota, Chinese Academy of Sciences, Xining, China; ^3^Technology Extension Service of Animal Husbandry of Qinghai, Xining, China; ^4^State Key Laboratory of Plateau Ecology and Agriculture, Qinghai University, Xining, China

**Keywords:** gut microbiota, high-altitude adaptation, 16S rRNA sequencing, meta-genomic sequencing, Tibetan wild asses

## Abstract

It was acknowledged long ago that microorganisms have played critical roles in animal evolution. Tibetan wild asses (TWA, *Equus kiang*) are the only wild perissodactyls on the Qinghai-Tibet Plateau (QTP) and the first national protected animals; however, knowledge about the relationships between their gut microbiota and the host's adaptability remains poorly understood. Herein, 16S rRNA and meta-genomic sequencing approaches were employed to investigate the gut microbiota–host associations in TWA and were compared against those of the co-resident livestock of yak (*Bos grunnies*) and Tibetan sheep (*Ovis aries*). Results revealed that the gut microbiota of yak and Tibetan sheep underwent convergent evolution. By contrast, the intestinal microflora of TWA diverged in a direction enabling the host to subsist on sparse and low-quality forage. Meanwhile, high microbial diversity (Shannon and Chao1 indices), cellulolytic activity, and abundant indicator species such as Spirochaetes, Bacteroidetes, *Prevotella_1*, and *Treponema_2* supported forage digestion and short-chain fatty acid production in the gut of TWA. Meanwhile, the enterotype identification analysis showed that TWA shifted their enterotype in response to low-quality forage for a better utilization of forage nitrogen and short-chain fatty acid production. Metagenomic analysis revealed that plant biomass degrading microbial consortia, genes, and enzymes like the cellulolytic strains (*Prevotella ruminicola, Ruminococcus flavefaciens, Ruminococcus albus, Butyrivibrio fibrisolvens*, and *Ruminobacter amylophilus*), as well as carbohydrate metabolism genes (GH43, GH3, GH31, GH5, and GH10) and enzymes (β-glucosidase, xylanase, and β-xylosidase, etc.) had a significantly higher enrichment in TWA. Our results indicate that gut microbiota can improve the adaptability of TWA through plant biomass degradation and energy maintenance by the functions of gut microbiota in the face of nutritional deficiencies and also provide a strong rationale for understanding the roles of gut microbiota in the adaptation of QTP wildlife when facing harsh feeding environments.

## Introduction

Adaptation is a core process in biological evolution and has attracted research attention since the time of Charles Darwin (Boyd, 2010). The microbial consortia in herbivore digestive tracts have co-evolved with their hosts. They convert lignocellulose hydrolysates into short-chain fatty acids (SCFAs) and play vital roles in host digestion (Kumar et al., 2021), health (Shreiner et al., 2015), immunity (Zhang et al., 2015), and adaptability (Ley et al., 2008). Advances in sequencing technology over the past decade have provided novel insights into microbiome co-evolution and adaptability. Furthermore, herbivore adaptation mediated via the gut microbiota has attracted a great deal of research attention (Liu et al., 2020d; Song et al., 2020; Fu et al., 2021).

The Qinghai-Tibet Plateau (QTP) is the highest plateau in the world, known as “the third pole” (Qu et al., 2020). It has special environmental conditions, such as high altitude, accompanied by low oxygen, severe cold, and high ultraviolet radiation. Yak (Y; *Bos grunniens*) and Tibetan sheep (S; *Ovis aries*) are the main indigenous livestock on the QTP. They have been grazed and used by local herdsmen for meat, milk, and fuel (the dry feces) for several centuries (Cui et al., 2020). Modification of the intestinal microbiomes of these high-altitude ruminants in response to the unique ecological environment of the QTP has been extensively studied (Ma et al., 2019b; Liu et al., 2020a,c, 2021; Fan et al., 2021). By way of illustration, S adapt to changes in the plant phenological period by altering their rumen microbial community structure and function (Liu et al., 2020a). Zhang et al. (2016) evaluated the production of SCFAs and emission of methane by *in vitro* rumen fermentation technique and found that Y accumulates high SCFAs concentrations in their rumen and attenuates methane emissions *via* microbial intervention, thereby harvesting energy more effectively than cattle. Tibetan wild ass (*Equus kiang*) is the only wild perissodactyl in the QTP. It has been endemic to the cold, hypoxic (4,000–7,000 m a.s.l.) montane, and alpine grassland for thousands of years, and often faces the problem of imbalance between herbage supply and the host's requirement (Schaller, 2000). Gut microbes are considered to be the link that converts herbage nutrients into the host's own nutrients; however, knowledge about whether these gastrointestinal microbes have special mechanisms to adapt to the ecological environments is limited. Only Gao et al. (2019) reported that Tibetan wild asses (TWA) adapt to the extreme environments of the QTP when their gut microbiota alter their fatty acid metabolism pathways, and Liu et al. (2020b) stated that TWA was superior to the domestic donkey in terms of gut microbiota community, function, and disease resistance under similar forage intake. Although the above work has described the structure and function of gut microbiota of TWA, the interaction between gut microbiota and the host and the response mechanism under environmental stress need to be further studied.

The Three-River-Source National Park is located in the hinterlands of the QTP and has a total area of 123,100 km^2^. It is the source place of the Yangtze, Yellow, and Lancang rivers. It is dominated by mountainous landforms with complex topographies and harbors vital habitats and biological germplasms of rare wild animal species (Qiao et al., 2018). As the awareness of ecological protection has increased in recent years, the number of wild animals such as TWA has increased to 36,000 in Three-River-Source National Park (Zhao et al., 2020). However, competition for forage often occurs between wild and domestic animals in this area. The overlapping habitats of wild and domestic animals in the region have enabled us to study the unique adaptive mechanisms of their intestinal microorganisms.

The large intestine, which included the cecum and the colon, harbored a higher bacterial diversity (Li et al., 2017) and is an important site of plant-fiber fermentation for asses. Analyses of fecal bacteria can characterize gut microbiota because the microorganisms in the feces resemble those in the large intestine (Zhao et al., 2015). In the present study, we used fresh feces from TWA, Y, and S as research objects and multi-omics methods to distinguish the unique gut microbial composition and high-altitude adaptation mechanisms of these animal groups. We speculate that there is a special microbial interaction mechanism in the gut of the TWA that is different from that of domestic animals. The aim of this study is to clarify the plateau adaptation mechanism of the TWA through their gut microbiota and provide a theoretical basis for investigations into the dietary adaptation of wild animals in QTP.

## Materials and Methods

### Sample Collection

**Figure 1:**
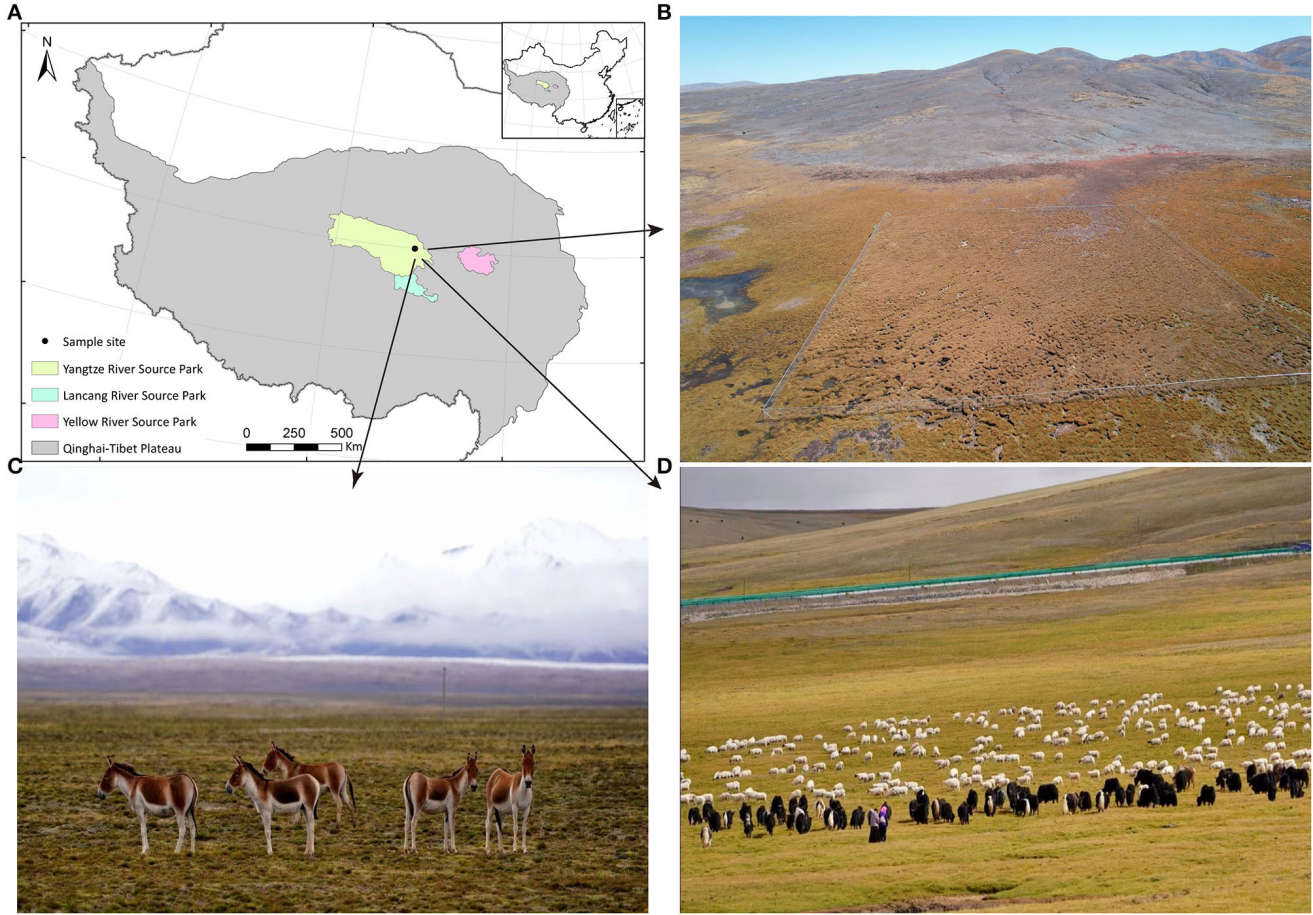
The information of sampling site and experimental animal habitat. **(A)** Sampling sites. **(B)** The aerial review of Three-River-Source Alpine Meadow Integrated System Observation in Yangtze River Source Park. The area is 100 × 100 m. **(C)** Small herds of TWA are leisurely feeding on the grass. **(D)** Y and S are feeding on abundant grass under the care of herdmen.

Fecal samples were collected on 13 August 2017 from co-resident TWA, S, and Y near the Three-River-Source Alpine Meadow Integrated System Observation in Yangtze River Source Park (Qumarleb County, Yushu Prefecture, Qinghai Province, China; Figure 1). The Yangtze River Source Park has an average altitude of 4,300 m, and the vegetation types are mainly alpine meadows, supplemented by desertified grasslands. Y and S in the area were under the supervision of herdsmen and usually foraged freely on grassland with abundant, high-quality herbage. As the human population is sparse and water sources were abundant in the area, wild animals such as TWA also foraged here. This condition created a sympatric living environment for all animals in the area. However, herdsmen's interference usually forced TWA to forage on nearby slopes with relatively little or low-quality vegetation.

As all animals dwelt in their natural habitat, they were highly alert. Therefore, the group was followed from a distance during sampling. After the animals defecated and left the area, feces were carefully but promptly collected from different individuals. To avoid cross-contamination, each sample was gathered with a disposable polyethylene glove that was used only once and then discarded. Twenty-seven fresh fecal samples were collected from seven S, nine Y, and 11 TWA. After collection, ~2 g of fresh fecal samples were placed in uniquely labeled cryotubes, frozen in liquid nitrogen, and stored at −80°C until subsequent high-throughput sequencing. Other samples (~50 g) were collected in sterile uniquely labeled zip lock bags, temporarily stored at 4°C in an automobile refrigerator, transferred to a laboratory refrigerator (4°C), and stored until subsequent apparent nutrient digestibility analyses. Fecal samples were collected within 1 d in their natural state. None of the animals received concentrate supplementation.

The herbage collection methods used here resembled those of Ma et al. (2019a). Briefly, ten 50 × 50 cm quadrats were randomly set at > 10-m intervals in an alpine meadow and the aboveground biomass was collected from them. To ensure that the forages collected were similar to those consumed by the animals, each quadrat was set on a forage with visible bite marks. Edible forage was collected, dried in a forced air stove at 60°C for 72 h, milled, passed through a 1-mm sieve, and stored in watertight plastic bags until subsequent dry matter (DM) digestibility and nutrition analyses.

### Chemical Analysis

The DM (oven method 930.15), crude protein (CP, Kjeldahl method 988.05), and ether extract (EE, diethyl ether extraction method 2003.5) contents were determined for forage–feces composites according to AOAC methods (AOAC, 2000). Acid detergent fiber (ADF) and neutral detergent fiber (NDF) were analyzed according to the methods of Van Soest et al. (1991). Nonfibrous carbohydrate (NSC) was calculated as follows:


NSC (%) = 100 − Ash (%) − NDF (%) − CP (%)

The nutrition of forage are shown in Supplementary Table 1.

### Determination of Host Apparent Nutrient Digestibility

Apparent nutrient digestibility was determined with acid-insoluble ash (AIA) as described by Liu et al. (2020b). Briefly, 5 g crushed forage and dried feces from the same individual were boiled in 50 mL of 4 N HCl for 30 min. The residue was filtered with a quantitative filter paper (*Φ*12.5 cm; NEWSTAR^®^, Hangzhou, China) and washed with hot distilled water until the filtrate was neutral. The residue and filter paper were ashed in a crucible in a muffle furnace (Beijing Zhongxing Weiye Instrument Co. Ltd., KSW-6-12) at 600°C for 12 h. After complete combustion of the organic matter, the crucible and ash were weighed on an analytical balance and the AIA values of the forage and feces were obtained. The total apparent CP, NDF, and ADF in the gut were calculated from the dietary and fecal nutrient. AIA ratios are as follows (Kavanagh et al., 2001):


$$\begin{array}{l} \text{Apparent digestibility }(\%)\text{ = 100 - 100 }\times \;({\text{AIA}}_{\text{h}}/AIA_{\text{f}})\\ \;\times \;({\text{Z}}_{\text{feces}}\;/\;{\text{Z}}_{\text{diet}}) \end{array}$$

where Z_feces_ and Z_diet_ are the nutrient concentrations (%) in the feces and forage, respectively, and AIA_h_ and AIA_f_ are the AIA concentrations (%) in the herbage and feces, respectively.

### Short-Chain Fatty Acids (SCFAs) Analysis

Short-chain fatty acids were detected according to the method of Fan et al. (2020). Briefly, acetic acid and propionic acid were measured by propyl chloroformate (PCF) derivatization followed by gas chromatography–mass spectrometry (GC-MS) (Zheng et al., 2013). Approximately, 0.1 g of each ~fecal sample was added to 1,000 μL of 0.005 M NaOH containing 5 μg/mL caproic acid–d3 internal standard (IS) and the suspension was homogenized. Then 500 μL of supernatant aliquot was transferred to a 15-mL capped centrifuge tube. Then 300 μL ultrapure water, 100 μL PCF, and 500 μL PrOH/Py solution (3:2, v/v) were added to the supernatant and the mixture was vortexed for 30 min. The derivatization included two extractions. In the first, 300 μL hexane was added to the mixture which was then vortexed for 1 min and centrifuged at 3,000 × *g* for 5 min. In the second, 200 μL hexane was added and 500 μL derivatized extract was collected in an autosampler vial and analyzed with an Agilent 7890A/5975C GC-MS (MSD; Agilent Technologies, Santa Clara, CA, USA).

### Determination of Cellulolytic Activity

Cellulolytic activity was determined based on the reaction between 3,5-dinitrosalicylic acid (DNS) and reducing sugar generated from a cellulose degradation assay (Miller, 1959). Gut contents (0.1 g) were placed in microcentrifuge tubes and homogenized in 1 mL extraction buffer at 4°C. The samples were homogenized on an ice bath and centrifuged at 8,000 × *g* and 4°C for 10 min. The supernatants were used in the enzymatic assays. Endo-β-1,4-glucanase activity was determined with a cellulase (CL) activity assay kit (Beijing Boxbio Science & Technology Co. Ltd., Beijing, China) according to the manufacturer's instructions. One unit of cellulolytic activity was defined as 1 g tissue producing 1 μg reducing sugar (glucose equivalent) per min.

### DNA Extraction and 16S rRNA Gene Illumina Sequencing

A fecal E.Z.N.A.^®^ DNA kit (Omega Bio-tek, Norcross, GA, USA) was used to extract the total microbial DNA from ~0.2 g fecal samples according to the manufacturer's protocols. The universal primers 341F (5′-barcode-CCTACGGGNGGCWGCAG-3′) and 806R (5′-GGACTACHVGGGTWTCTAAT-3′) were used to amplify the bacterial V3–V4 hypervariable region (Liu et al., 2020b). The barcodes were eight-base sequences unique to each sample. A 30-μL PCR amplification mixture was prepared for each sample and consisted of 15 μL of 2 × Phanta Master Mix, 1 μL of each primer (10 μM), and 20 ng template DNA. The cycling parameters for the PCR amplification included an initial denaturation at 95°C for 5 min followed by 30 cycles at 95°C for 30 s, annealing at 55°C for 30 s, elongation at 72°C for 45 s, and a final extension at 72°C for 5 min. All PCR products were detected on 2% agarose gels and purified with an AxyPrep DNA gel extraction kit (Axygen Biosciences, Union City, CA, USA) according to the manufacturer's instructions. They were then quantified with QuantiFluor™-ST (Promega, Madison, WI, USA). The sequence library was generated with a Qubit^®^ 3.0 fluorometer (Invitrogen, Carlsbad, CA, USA) and an Agilent Bio Analyzer system. The amplicons were pooled into equimolar sample concentrations. After quality assessment, the library was sequenced on an Illumina HiSeq 2500 platform (Nanjing Gene Pioneer Co. Ltd., Nanjing, China) and 250-bp paired-end reads were generated.

To obtain high-quality sequences, raw reads were quality-filtered with QIIME v. 1.1.7 (https://qiime2.org; Caporaso et al., 2010). All sequences were trimmed and assigned to each sample based on their unique barcodes and primers. The criteria were: barcode mismatches = 0 and maximum primer mismatches = 2. Overlapping paired-end reads were merged with PANDAseq v. 2.10 (https://github.com/neufeld/pandaseq; Masella et al., 2012). Merged sequences with high-quality reads (read length > 300 bp, no ambiguous base “N,” and average base quality score > 30) were used in the subsequent analyses. Metaxa2 (https://microbiology.se/software/metaxa2/) was used to remove potential chloroplast sequences from PCR amplification (Bengtsson-Palme et al., 2015). Singleton and chimeric reads were removed with UCHIME (https://www.drive5.com/uchime/uchime_download.html; Edgar et al., 2011). Operational taxonomic units (OTUs) were selected with UPARSE v. 7.1 (https://github.com/wmaier/uparse), abundance-based greedy clustering, and a 97% identity threshold. The OTUs were annotated with Ribosomal Database Project classifier v. 2.2 (rdp.cme.msu.edu). To compare samples with different sequencing depths, random sequence number leveling (38,327 reads) was performed on each sample with the smallest sequence number. Alpha diversity indices including Shannon diversity, Chao1, Good's coverage, and PD whole tree were calculated.

### Enterotype Clustering

Enterotype clustering is widely used in humans (Arumugam et al., 2011; Costea et al., 2018). Genus-level relative abundance profiles of the samples were clustered by Jensen-Shannon divergence (JSD), B-C dissimilarity, and partitioning method (PAM) clustering in R v. 4.1.2 (R Core Team, Vienna, Austria). The Calinski-Harabasz (CH) index and the silhouette score were used to evaluate cluster robustness (Hildebrand et al., 2013). The PAM, CH index, and silhouette score were then applied to cluster enterotypes by the B-C dissimilarity and JSD methods, and the same results were obtained by both the methods (Supplementary Figure 1). Earlier studies suggested that B-C dissimilarity is strongly correlated with JSD (Arumugam et al., 2011; Guo et al., 2021) and revealed variations in the abundance of taxa with enterotypes. Hence, B-C dissimilarity was conducted at the genus level. The similarity percentage (SIMPER) method (https://rdrr.io/rforge/vegan/man/simper.html) was used to identify and rank the contribution of each genus among the enterotype groups (Clarke, 1993).

### Shotgun Meta-Genomic Sequencing, Assembly, and Annotation

Sixteen samples were selected (TWA, *n* = 7; Y, *n* = 4; S, *n* = 5) for meta-genomic sequencing. Genomic DNA quality and purity were assessed with a NanoPhotometer^®^ spectrophotometer, a Qubit^®^ dsDNA assay kit, and a Qubit^®^ 2.0 fluorometer (Life Technologies, Waltham, MA, USA). Genomic DNA with OD in the range of 1.8–2.0 and mass > 1 μg was used as the input for sequencing library generation with a NEBNext^®^ Ultra™ DNA library prep kit (New England Biolabs, Ipswich, MA, USA). Sequences with index codes were added to split each sample. The samples were sonicated to generate ~350-bp DNA fragments, which were then end-polished, A-tailed, and ligated with full-length adaptors for PCR amplification. The PCR products were cleaned and extracted in an AMPure XP system (Beckman Coulter, Brea, CA, USA). Libraries were prepared in a cBot cluster generation system (Illumina, San Diego, CA, USA) according to the manufacturer's protocols. After cluster generation, the prepared library was sequenced on an Illumina HiSeq 2500 platform (Novogene Biological Information Technology Co., Beijing, China), and 150-bp paired-end reads were generated.

To get high-quality clean reads, sequences with low base quality (quality value < 38%; > 40 bp) and N bases > 10 bp were removed to obtain high-quality clean reads. Bowtie2 (https://www.encodeproject.org/software/bowtie2/) was used to remove reads contaminated by adaptor and host sequences (Karlsson et al., 2012, 2013; Scher et al., 2013). The configuration parameters were: –end-to-end, –sensitive, -I 200, and -X 400. After filtering, clean data with k-mers = 55 were assembled into scaftigs with SOAPdenovo (https://github.com/aquaskyline/SOAPdenovo2; Luo et al., 2012) using the following configuration parameters: -d 1, -M 3, -R, -u, and -F. N50 length was used to evaluate the quality of the assembly. MetaGeneMark v. 2.1.0 (https://github.com/gatech-genemark/MetaGeneMark-2; Nielsen et al., 2014; Oh et al., 2014) was used to predict the contigs (≥500 bp) in each sample. Open reading frames (ORFs) derived from the assembled contigs were clustered into a non-redundant dataset with CH-HIT (Li and Godzik, 2006). The configuration parameters were: -c 0.95, -G 0, -aS 0.9, -g 1, and -d 0. Bowtie2 was used to count the reads generated from the re-aligned reads and reduce the number of redundant genes. A gene catalog was obtained from the nonredundant genes with > 2 reads. After read filtering, DIAMOND (https://github.com/bbuchfink/diamond; Buchfink et al., 2015) was used to align the unigenes with bacteria and archaea extracted from the NR database v. 2018.01 (National Center for Biotechnology Information (NCBI), Bethesda, MD, USA; blastp; *e*-value ≤ 1e-5) for taxonomic profile annotation. For gene function annotation, the unigenes were blasted with the CAZy, KEGG, and eggNOG functional databases in DIAMOND.

### Statistical Analysis

Standard R commands were performed to identify the differences in relative abundance across groups. Kruskal–Wallis (multiple groups) and Dunn's *post-hoc* (two-sample comparisons) tests were used to evaluate significant differences in the nonparametric profiles. The Mann–Whitney *U*-test was used to calculate the contributions of the gut microbiota associated with the various enterotypes. R v. 4.0.2 generated Sankey plots or boxplots to visualize the relative abundances of the microbial taxa and concentrations of SCFAs across hosts. B-C dissimilarity and nonmetric multidimensional scaling (NMDS) were run in the *vegan* package v. 2.5-7 of R (Oksanen et al., 2013). To identify the microbial taxa that best represented variations in this parameter across hosts, an indicator analysis was conducted in the *indicspecies* package v. 1.7.9 in R. Multiptt function with 9,999 permutations was performed on the list of species correlated with a group of samples. The r.g. function was used to determine the correlations between the binary vectors (Guo et al., 2021). A Mantel test with 9,999 permutations calculated the correlations between gut environmental factors and microbial community data and was run in the linkETv. 0.0.3.3 package of R (https://github.com/Hy4m/linkET), and was based on calculated environmental (Euclidean) and taxonomic (Bray-Curtis) distances. Pairwise Pearson's correlation analyses were implemented using the “quickor” function in *ggcor* to evaluate the significance of the relationships among environmental parameters. Heat maps of the glucoside hydrolase family genes were generated with the pheatmap v. 1.0.12 package in R.

## Results

### Forage Apparent Nutrient Digestibility and Cellulytic Activity

As shown in Table 1, the apparent nutrient digestibility of CP in the TWA group (81.26%) was significantly higher than that of S (*P* < 0.05), but not significantly with Y. The cellulytic activity in the TWA group (517.19 U/g) was significantly higher than that of Y and S (*P* < 0.05). No significant differences were detected for DM (*P* = 0.215), NDF (*P* = 0.171), and ADF (*P* = 0.313) digestibility among the three groups.

**Table 1:** The apparent nutrient digestibility and cellulytic activity across different hosts.

**Items**	**Groups**			***P*-values**
	**Y**	**S**	**TWA**	
Apparent nutrient digestibility (%)				
DM^a^	69.20 ± 4.89	72.16 ± 3.33	67.16 ± 9.51	0.215
CP	76.92 ± 2.11^a^	70.46 ± 3.65^b^	81.26 ± 4.38^a^	<0.05
NDF	62.27 ± 7.33	59.19 ± 1.16	65.14 ± 3.37	0.171
ADF	52.14 ± 2.74	51.13 ± 1.05	54.63 ± 5.75	0.313
Cellulytic activity(U/g)	450.66 ± 14.39^b^	436.45 ± 12.26^b^	517.79 ± 10.02^a^	<0.05

a *DM, dry matter;*

*CP, crude protein; NDF, netural detergent fiber; ADF, acid detergent fiber. In the same row, values with different letters mean significant different (P < 0.05), while with the same letter or no superscripts mean no significant difference (P > 0.05)*.

### Similarity and Dissimilarity of Gut Bacteria in Co-resident Wild and Domestic Animals

**Figure 2:**
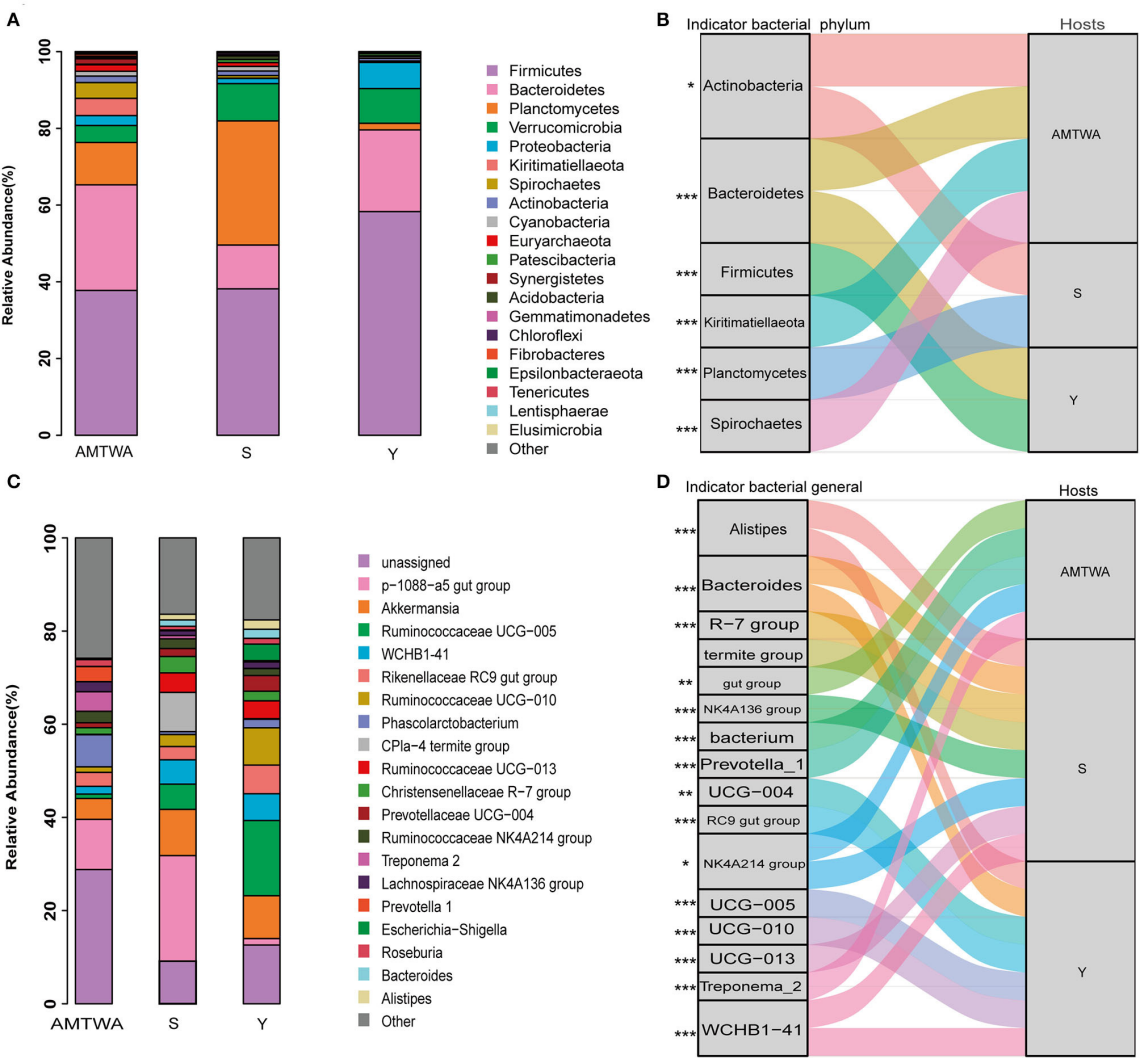
The relative abundances of gut microbiota and indicator species among animal hosts. Relative abundances of the 20 most abundant phyla **(A)** and genera **(C)** in different hosts are aggregated and colored on stack graphs. Low abundance taxa are grouped together and labeled “other.” Indicator phyla **(B)** and genera **(D)** related to each hosts are traced by Sankey plots. Line represent associations between indicator taxa and hosts. Different colors represent different taxa. Line width is scaled to reflect indicator value. Higher indicator values for each taxon are more strongly associate with different hosts. Indicator values are shown in Supplementary Figure 2. The genera *R-7 group, termite group, gut group, NK4A136 group, bacterium, UCG*-*004, RC9 gut group, NK4A214 group, UCG-005, UCG*-*010* and *UCG*-*013* are represent *Christensenellaceae R-7 group, CPla-4 termite group, p-1088-a5 gut group, Ruminococcaceae NK4A136 group, Phascolarctobacterium, Prevotellaceae UCG-004, Rikenellaceae RC9 gut group, Ruminococcaceae NK4A214 group, Ruminococcaceae UCG-005, Ruminococcaceae UCG-010* and *Ruminococcaceae UCG-013*, respectively. The statistical *P-*values mean the taxa associated with hosts. *P < 0.05, **P < 0.01, ***P < 0.001.

Sequencing of the 16S rRNA amplicons yielded 2,178,233 high-quality sequences, 1,858,891 effective sequences after singleton removal, and 29,631 operational taxonomic units (OTUs) (Supplementary Table 2). Twenty gut bacteria phyla were identified, and Firmicutes (44.22% of the total on average), Bacteroidetes (20.60%), and Planctomycetes (15.10%) were the most abundant in all the hosts. Together, the foregoing three phyla accounted for ~80% of the entire fecal microbiota (Figure 2A, Supplementary Table 3). The similarity of gut flora was also exhibited at the genus level in both TWA and domestic animals, with *p-1088-a5 gut group* (11.61% of total average), *Akkermansia* (7.85%), and *Ruminococcaceae UCG-005* (7.51%) the most abundant across the hosts (Figure 2C, Supplementary Table 4).

**Figure 3:**
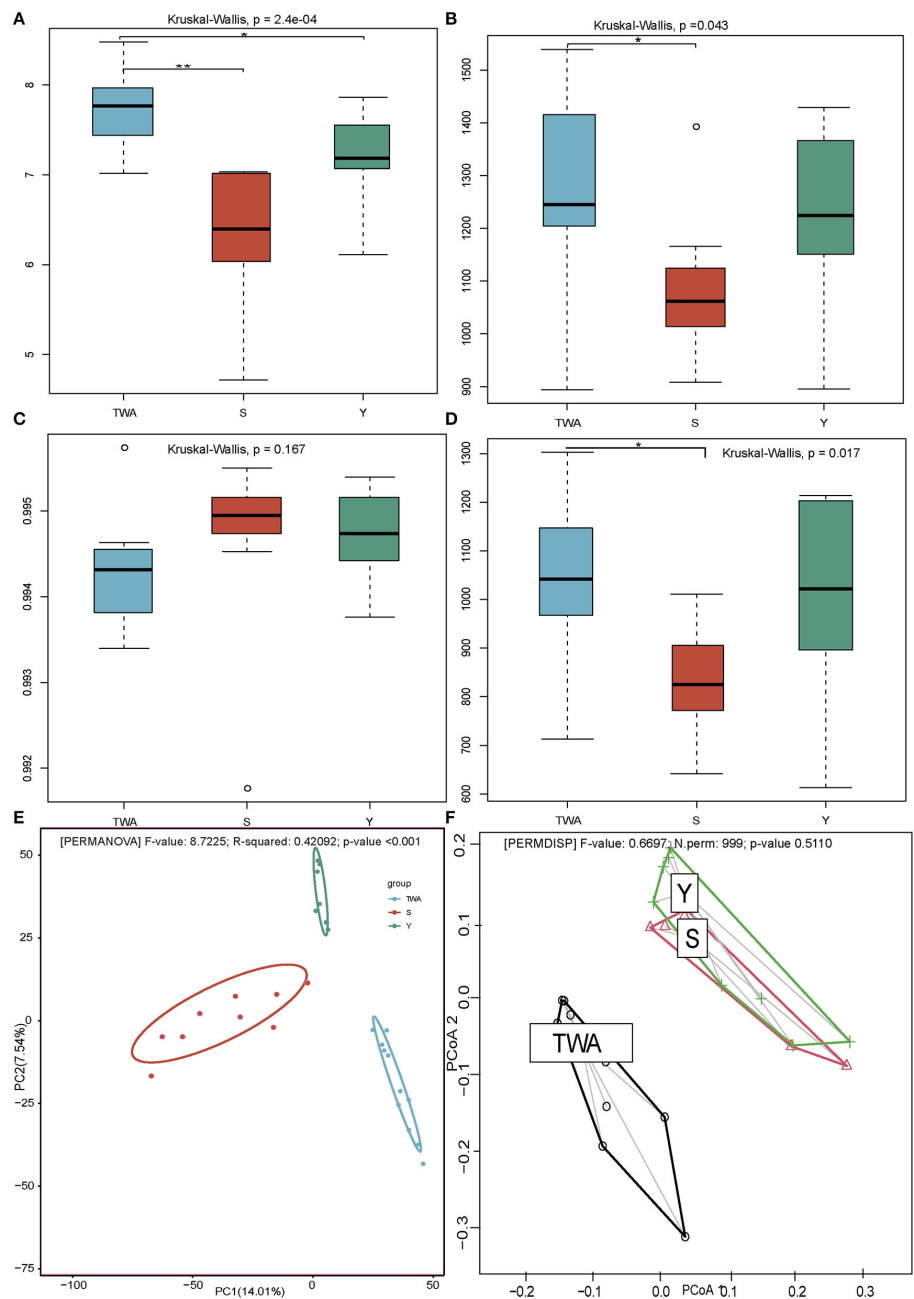
Diversity metrics of gut microbiota communities among TWA, S, and Y. Shannon diversity **(A)**, Chao1 index **(B)**, Good's coverage **(C)**, and PD whole **(D)** indices represented by boxplots. PCoA **(E)** plots show distributions of samples based on Bray-Curtis distance matrix. Significant differences are evaluated by PERMANOVA with *P* < 0.01. Percent variation explained by each principal coordinate is indicated next to corresponding axis. Homogeneity of dispersions **(F)** for this diversity metric is also tested to identify differences in variance among treatments. Significant differences are determined using 999 permutations of the betadisper function in the vegan v. 2.5-6 package of R. All boxplots distribution are tested by non-parametric Kruskal–Wallis and Dunn *post hoc* tests with FDR-corrected *P*-value, boxplots center values indicate the median, and whiskers represent 0.75 times the interquartile range. * < 0.05, ** < 0.01, no * mean no significant difference.

The dissimilarity was manifested in alpha and beta diversity. The Shannon diversity in TWA was significantly higher than that in Y and S (*P* = 0.0002, Figure 3A), meanwhile, the Chao1 index in TWA was significantly higher than S (*P* = 0.043, Figure 3B) as well, which indicated that the gut microbiota communities in TWA were more plentiful in diversity. No significant differences in good coverage index (*P* = 0.167, Figure 3C) indicated that the samples of the bacterial community were fully sampled. The phylogenetic diversity metrics (PD whole tree) analysis (*P* = 0.017, Figure 3D) showed the gut flora of S and Y had a closer evolutionary relationship, whereas, TWA had obvious community differentiation. Moreover, the beta diversity analysis by using PCoA (Figure 3E) and the homogeneity of dispersions test (Figure 3F) revealed that the composition of the gut prokaryotic community among the TWA, Y, and S were significantly different, and the gut microbiota structure of TWA differs from that of domestic animals.

Indicators analysis at the phylum and genus level also displayed differences across different groups. In the S group, the indicator species at the phylum (Figure 2B) and genus (Figure 2D) levels were *Actinobacteria, Planctomycetes, Alistipes, Bacteroides, Christensenellaceae R-7 group, CPla-4 termite group, and Lachnospiraceae NK4A136 group*. In the Y group, the indicators were *Bacteroidetes, Firmicutes, Alistipes, Bacteroides, Prevotellaceae UCG-004, Rikenellaceae RC9 gut group, Ruminococcaceae UCG-005, Ruminococcaceae UCG-010, and WCHB1-41*. In the TWA group, they were *Actinobacteria, Bacteroidetes, Kiritimatiellaeota, Spirochaetes, p-1088-a5 gut group, Phascolarctobacterium, Prevotella_1, Rikenellaceae RC9 gut group, Ruminococcaceae NK4A214 group*, and *Treponema 2*. Notably, indicators (phylum *Spirochaetes* and *Bacteroidetes*; genus *Prevotella_1* and *Treponema_2*) which had the higher indicate value had a higher relative abundance in the TWA group (Supplementary Figure 2, Supplementary Tables 3, 4).

### SCFAs Profiles and Their Correlations With Gut Microbiota Communities

**Figure 4:**
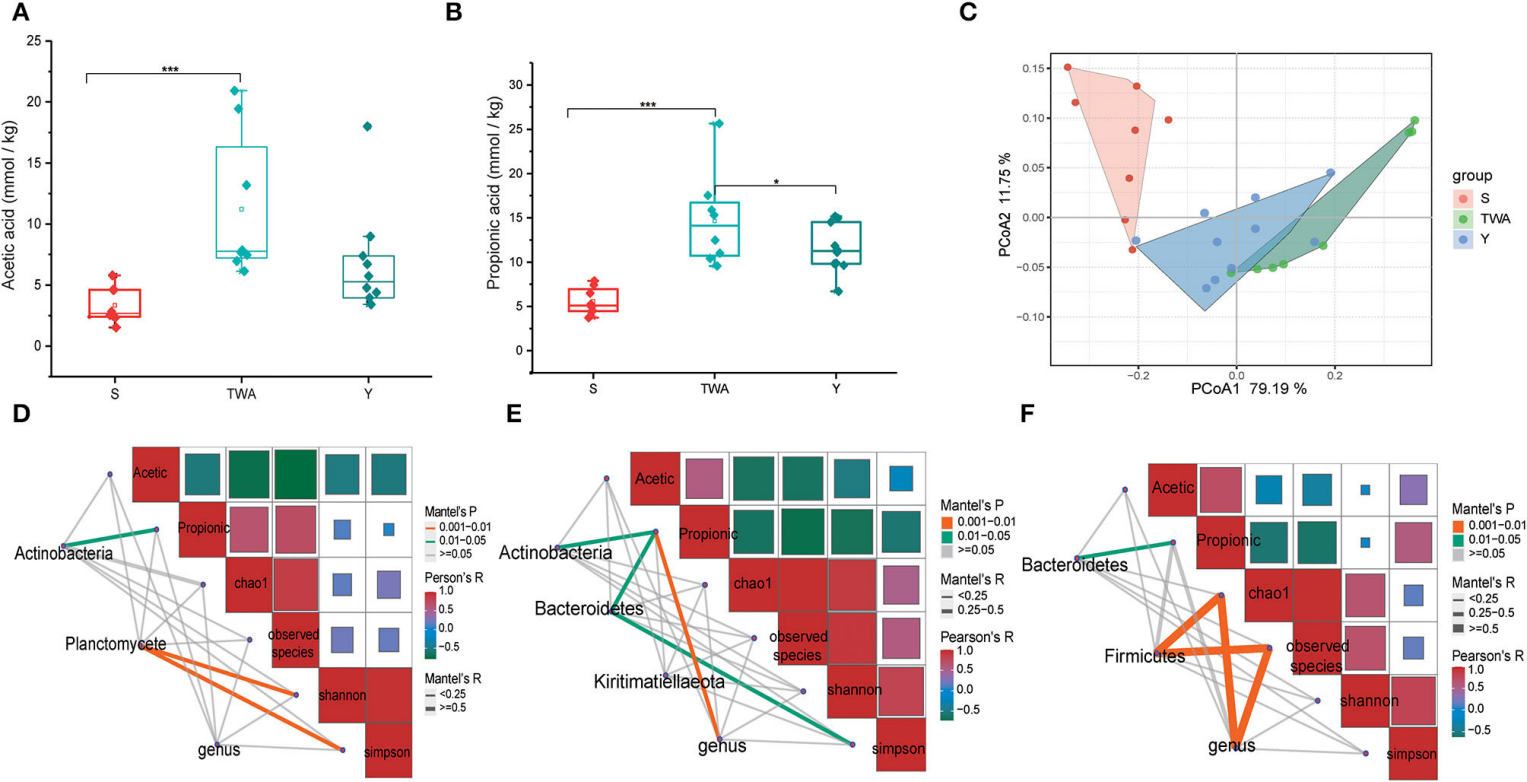
The concentrations of dominant SCFAs and correlations among bacteria, SCFAs concentration, and gut microbiotia diversity in the three different animal hosts. **(A)** Acetic acid concentration. **(B)** Propionic acid concentration. **(C)** PCoA of SCFAs profile based on Bray-Curtis distance between host species. **(D–F)** Are the correlation analysis among bacteria, SCFAs concentration, and gut microbiota diversity in S, TWA, and Y, respectively. ***P < 0.001, *P < 0.05.

We surveyed two dominant SCFAs, and the results showed that both the concentration of acetate acid (Figure 4A) and propionate acid (Figure 4B) in the fecal of TWA were significantly higher than S (*P* < 0.001), and the propionic acid concentration was significantly higher than Y as well (*P* < 0.05). These results were consistent with PCoA analysis, in which the SCFAs profile in TWA had a trend to congregate with Y, on the contrary, separated with S (Figure 4C).

To further explore the driving factors affecting the production of SCFAs, we performed a correlation analysis using the indicator species phyla and genera as the driving factors and SCFAs and bacterial alpha diversity as the environmental factors. In the S group, *Actinobacteria* have positive effects on propionic acid production, and bacterial community diversity was affected by *Planctomycete* (Figure 4D). In the TWA group, the relative abundance of *Actinobacteria, Bacteroidetes*, and genus community has positive effects on propionic acid production, and Simpson indices was affected by *Bacteroidetes* (Figure 4E). In the Y group, *Bacteroidetes* had positive effects on propionic production, and both *Firmicutes* and genus have significant positive effects on bacterial richness (Figure 4F).

### Gut Enterotypes and Functional Contexts of *Treponema 2, WCHB1-41*, and *Prevotella 1* in the Adaptation to Sparse Vegetation

**Figure 5:**
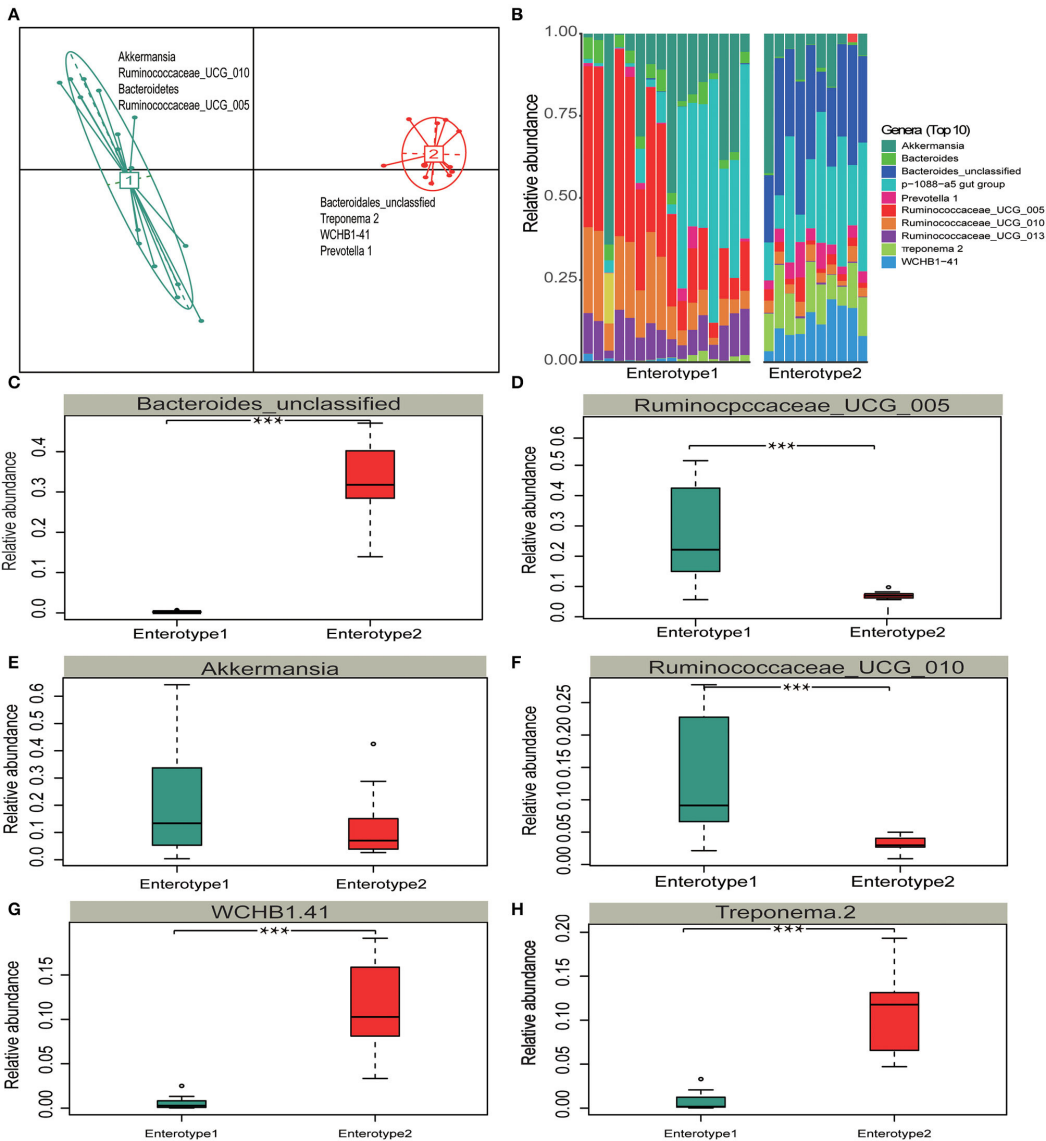
Enterotype distributions of gut microbiota associated with different hosts using Bray-Curits dissimilarity. Identification of gut enterotypes is shown in Supplementary Figure 1. **(A)** Visualizations of enterotypes, indentified by PAM (partitioning around medoid) clustering. **(B–H)** Relative abundances of bacterial taxa characteristic of each enterotype. Ten genera were selected based on their average contributions to the overall Bray-Curtis dissimilarity. All four bacterial genera are shown in Supplementary Figure 3. All boxplots distributions are test by Mann-Whitney *U*-test with FDR-corrected *P*-value, box-plots center values indicate the median, and whiskers represent 0.75 times the interquartile range. *** < 0.001, no * mean no significant difference.

Different enterotypes may functionally differ (Costea et al., 2018). Therefore, we investigated whether the gut microbiota partition into clusters with functional properties that vary among hosts. A principal component analysis (PCA) revealed that samples formed two distinct enterotype clusters based on their BC dissimilarities. The intestinal microflora of Y and S belonged to the enterotype1 clusters whereas the intestinal microflora of TWA belonged to the enterotype2 clusters. The clusters were distinguished by the relative differences in their representative bacterial genera. *Akkermansia, Ruminococcaceae_UCG_010*, Bacteroidetes, and *Ruminococcaceae_UCG_005* were in enterotype1 while *Treponema 2, WCHB1-41*, and *Prevotella* 1 were in enterotype2 (Figure 5A). The top 10 relative abundance of representative bacterial genera (Figure 5B) of *Ruminococcus_UCG_005, Ruminococcaceae_UCG_010, Akkermansia, Ruminococcaceae_UCG_013*, and *Bacteroidetes* had significantly higher enrichment in enterotype1 (*P* < 0.001; Figures 5C–H, Supplementary Figure 3). In contrast, the relative abundance of *Bacteroides_unclassified, WCHB1-41, Treponema 2*, and *Prevotella _1* were significantly higher in enterotrpe 2 (*P* < 0.001).

**Figure 6:**
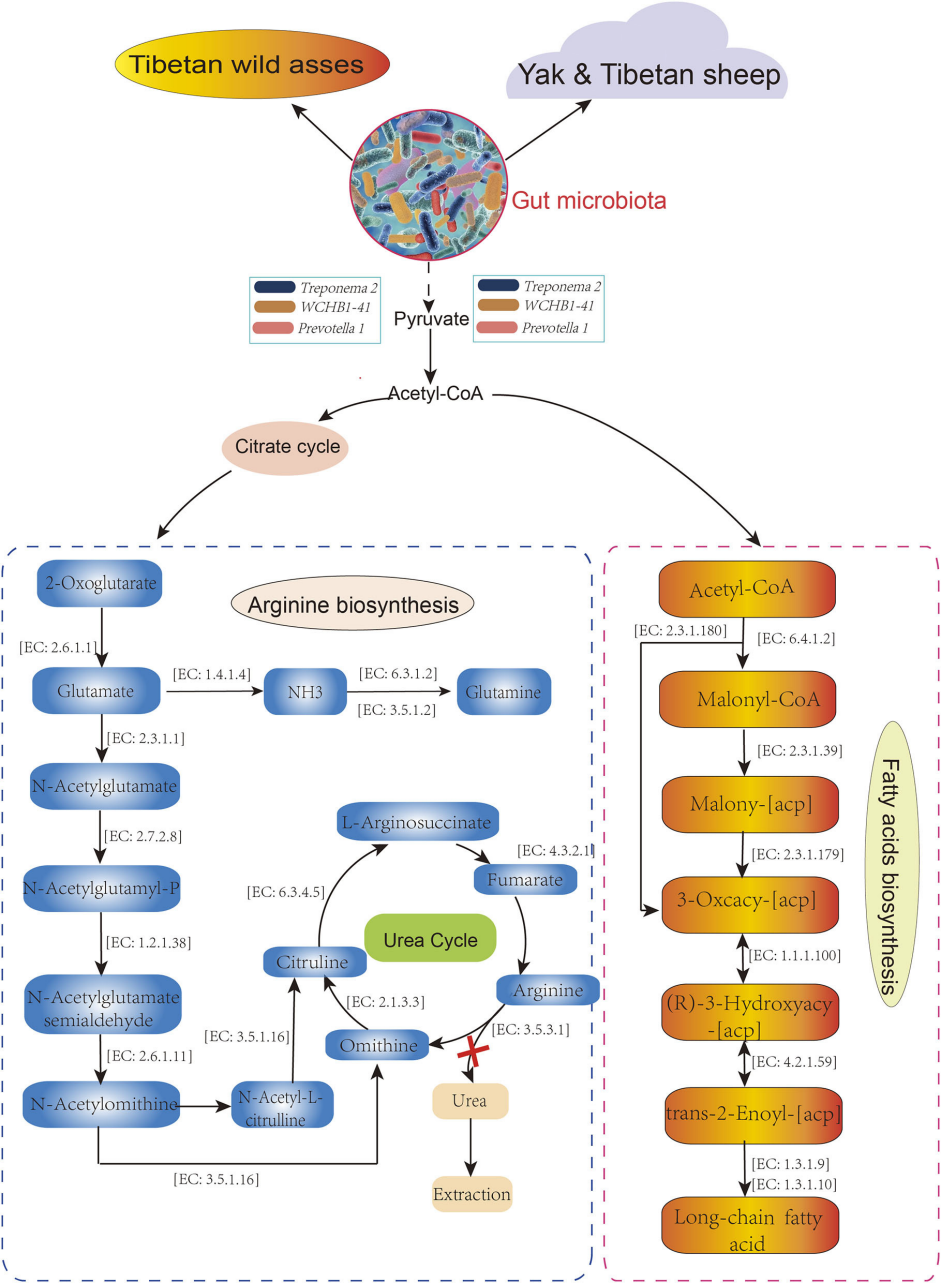
Metabolic pathways in host associate with *Treponema 2, Prevotella 1*, and *WCHB1-41*. All enzymes and EC numbers are obtained from Kyoto Encyclopedia of Genes and Genomes (KEEG) database.

The distributions of the gut enterotypes in these hosts led us to hypothesize that the fixed enterotype represented by *Treponema* 2, *WCHB1-41*, and *Prevotella 1* regulates the assimilation of essential nutrients from sparse forage. To test the hypothesis, we queried the functional relevance of *Treponema 2, Prevotella_1*, and *WCHB1-41* in the Kyoto Encyclopedia of Genes and Genomes (KEGG) database. *Treponema* 2, *WCHB1-41*, and *Prevotella_1* showed convergent enrichment of the enzymes involved in the arginine (Arg; map: 00220) and fatty acid (FA) biosynthesis (map: 00061) pathways (Figure 6). Pyruvate oxidation and decarboxylation to acetyl CoA are mediated by *Treponema 2, Eubacterium*, and *WCHB1-41*, and they enter the citrate cycle, promote Arg biosynthesis, and regulate FA biosynthesis. We detected 14 enzymes involved in Arg biosynthesis but no enzyme regulating urea synthesis. This physiological configuration mitigated urinary N loss in TWA under low-N stress. Seven of the eight foregoing enzymes directly participated in FA biosynthesis and energy generation. The preceding results suggested that both the Arg and FA biosynthesis pathways evolved in the gut of TWA grazing on sparse vegetation to ensure that they utilize N and generate energy under these conditions.

### Meta-Genomic Sequencing Profile and the Functional Microbita Related to Cellulose Degrading and Methane Producing

Sixteen samples were selected (TWA, *n* = 7; Y, *n* = 4; S, *n* = 5) based on their β-diversity and Bray-Curtis (BC) distance values (Figure 3E) for meta-genomic sequencing. About 12.40 G clean bases per sample (Supplementary Table 5), with an average N50 length of 1.47 kb, including 14.93 million non-redundant unigenes and an average ORF length of 653 bp. A total of 3,766,521 genes were cataloged in the NR database annotation. The overall and domain-, phylum-, genus-, and species-level annotation rates were 80.75, 75.38, 49.38, and 34.31%, respectively (Supplementary Table 6).

**Figure 7:**
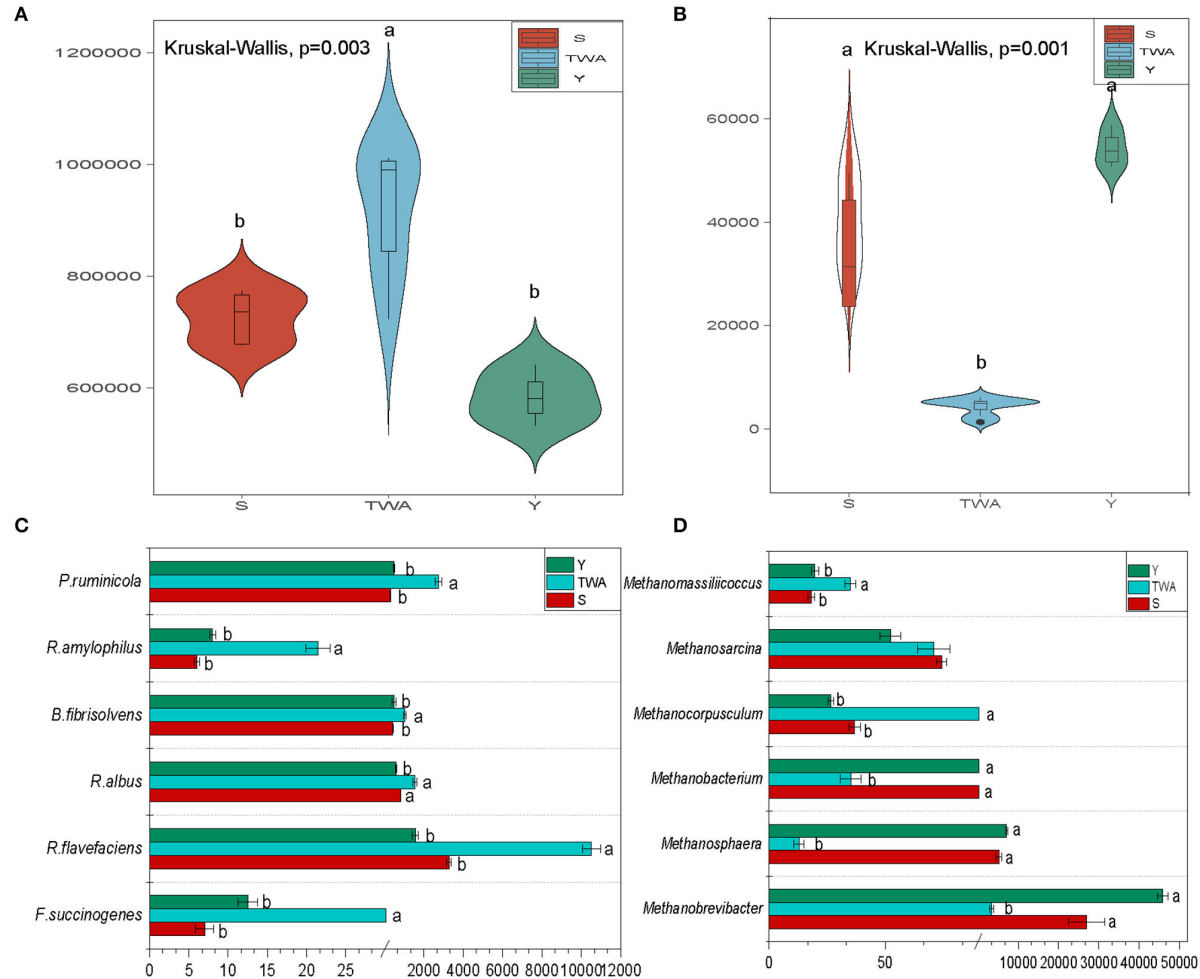
Gut microbiota composition, and functional microbiota relate to cellulose degradation and methane production. Violin plot showing relative abundances of bacteria **(A)** and archaea **(B)** in different hosts. Analysis of relative abundances of cellulose-degrading species **(C)** and methane-producing archaea **(D)**. Different letters denote statistically significant differences at corrected *P* < 0.05.

The bacterial abundance was significantly higher (*P* = 0.003) in the gut of TWA than it was in those of S and Y (Figure 7A). By contrast, the abundance of archaea was significantly lower (*P* = 0.001) in the gut of TWA than it was in those of S and Y (Figure 7B). We then analyzed gut microbiota related to cellulose decomposition and methane production. The abundances of the cellulolytic bacteria *Prevotella ruminicola, Ruminococcus flavefaciens, Ruminococcus albus, Butyrivibrio fibrisolvens*, and *Ruminobacter amylophilus* were significantly higher in the gut of TWA than they were in those of Y and S (Figure 7C, Supplementary Table 7). *Methanobrevibacter* and *Methanosphaera* were the predominant methanogenic archaea in all samples, but their abundances were significantly higher in TWA than Y or S (Figure 7D, Supplementary Table 7).

### Carbohydrate-Metabolizing Genes

**Figure 8:**
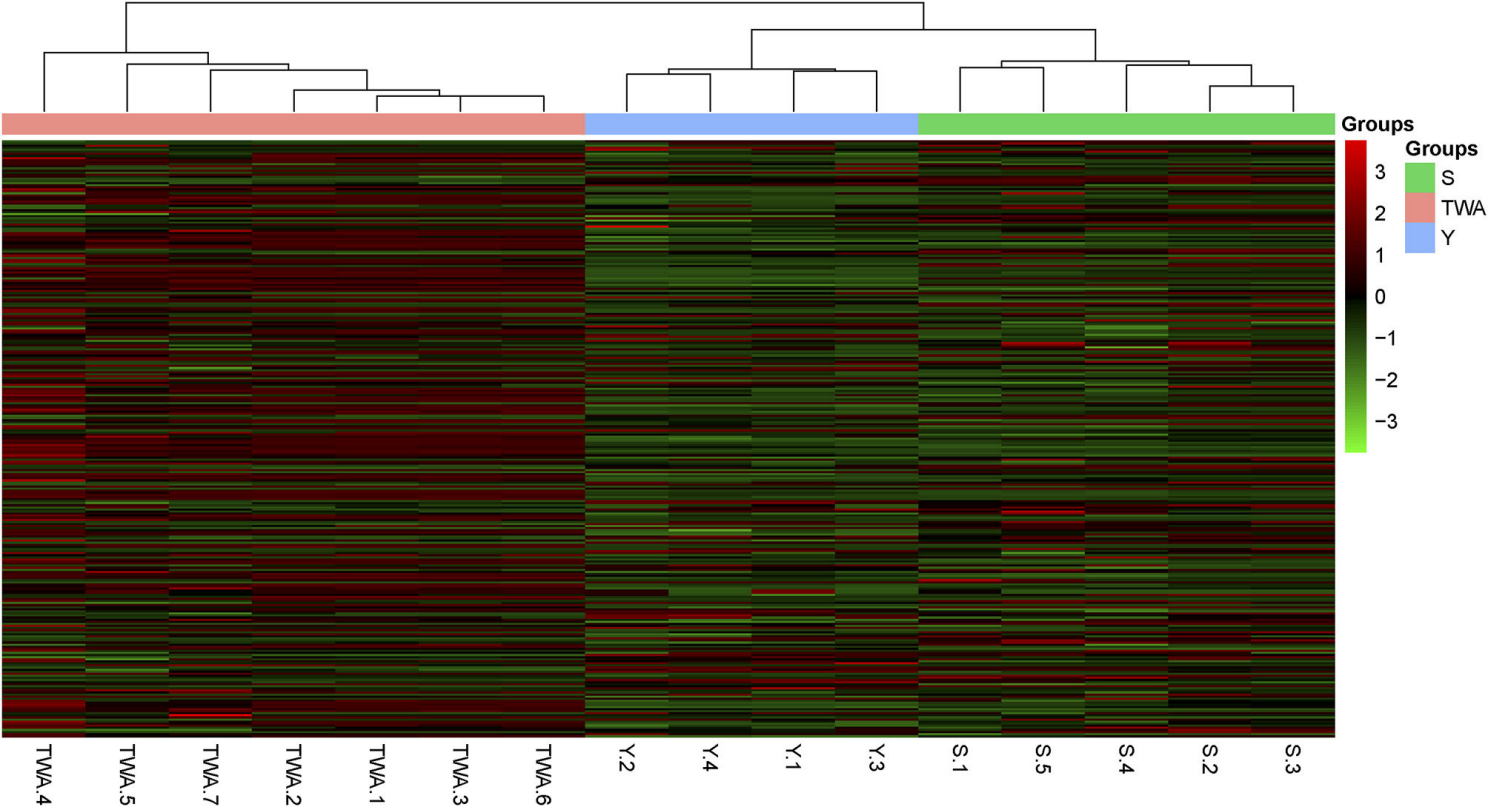
Heatmap visualization of the distribution of non-redundant genes among samples. Heatmap based on hierarchical clustering solution (Euclidean distance) of 16 samples. Rows represents 286 glucoside hydrolase family genes. Columns represent 16 samples. Relative abundance of each gene is normalized [log2 (data+1)] and displayed in rows of cells.

To establish the potential capacity of gut bacteria to degrade herbage biomass, we sought carbohydrate-active enzymes (CAZymes) among the nonredundant genes. A hierarchically clustered heatmap (Figure 8) based on the overall CAZyme families disclosed that the unigenes in S and Y clustered together and separated from those in TWA. The compositions of the unigenes in Y and S resembled each other more closely than those of the unigenes in TWA.

**Figure 9:**
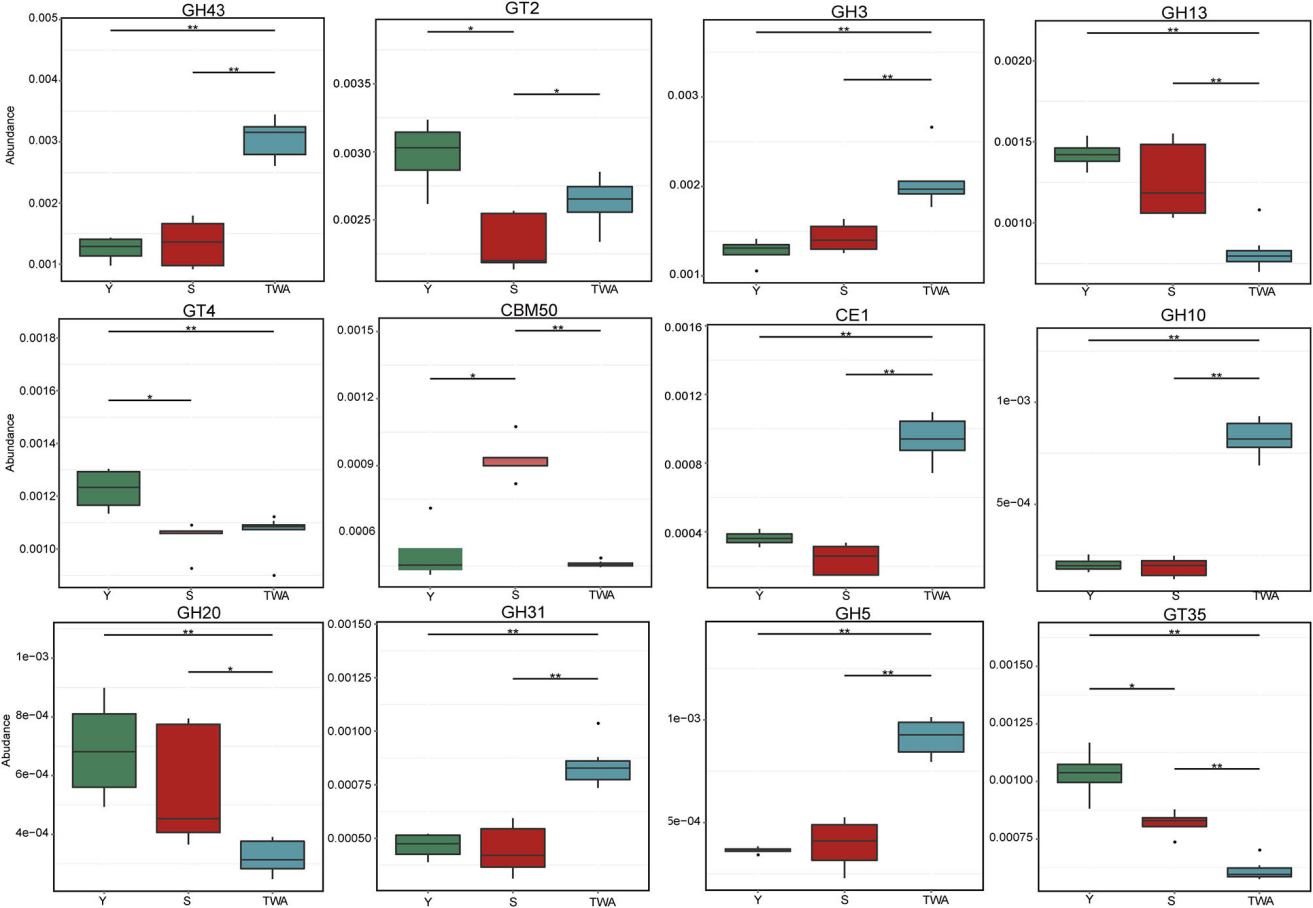
Relative abundances of top12 genes encoding glycoside hydrolases (GHs), glycosyltransferases (GTs), carbohydrate esterases (CEs), and carbohydrate-binding moudles (CBMs). *P < 0.05, ^**^P < 0.01.

To further explore the significance of these unigenes, a metastat analysis identified the underlying CAZyme families and the EC numbers (top 12 in terms of relative abundance) for the constituents differing among animal groups. In addition to GT2, the CAZymes GH43, GH3, GH31, GH5, and GH10 associated with cellulose and hemicellulose degradation were significantly more abundant in TWA than Y or S. Conversely, GH13, GH20, CE1, GT35, and GT4 related to starch and carbohydrate ester decomposition were significantly less abundant (*P* < 0.05) in TWA than Y or S (Figure 9, Supplementary Table 8). All the EC numbers in TWA were significantly higher than those in Y and S (*P* < 0.05; Supplementary Figure 4).

## Discussion

The low temperatures and hypoxia of the QTP make it an extremely harsh environment for mammalian species. Y, S, and TWA are primary consumers of alpine grassland. They graze the QTP grasslands all year and must contend with the severe challenges of extreme cold and limited food availability. Especially for TWA, they receive no special care such as forage supplementation from herdsman (Liu et al., 2019) and must subsist on sparse native forage. In the present study, we considered the nutrition and apparent digestibility of forage and cellulose activity in three co-resident herbivore species. In general, monogastric animals less effectively digest and assimilate high-fiber, low-protein grass than ruminants such as Y and S (Hintz et al., 1978). The high apparent digestibility of herbage is one of the vital factors for Y and S to adapt to the long cold season (~6 months) and seasonal imbalance of pasture production of QTP (Dong et al., 2006). Similarly, forage digestibility of TWA determines the host's ability to cope with the nutrient stress of forage. In the present study, the higher apparent digestibility of TWA in CP (81.26%), NDF (65.14%), ADF (54.63%), and cellulose activity (517.79 U/g) revealed that TWA had a relatively more efficient nutrient uptake and could, therefore, withstand harsher nutritional stress than the domestic animal.

Host forage digestibility depends on the synergistic action of various gut microbiota (Brune and Ohkuma, 2010; Gomez et al., 2021). Host genome, phylogeny, diet, and environmental heterogeneity are usually the main factors affecting gut microbial community structure (Grieneisen et al., 2019). In our study, Y and S are the different species from different genera; however, there was no significant difference in their gut microbial diversity. This indicated that host genetics may not be a decisive factor influencing gut microbiota composition (Martinson et al., 2017). Host phylogeny can influence the microbial community diversity through immune-related genes that shape host–microbita interactions (Zhang et al., 2013, 2015). In our study, the experimental animals belonged to different phylogenies, and thus the different gut microbiota diversity and community structure might be related to this. However, based on a previous study of gut microbiota of captive colobine monkey species, researchers found that colobine phylogenetic relationships was not reflected in the gut microbiota (Vanessa et al., 2017); therefore, we speculated that phylogeny may not be a decisive factor as well. Of course, the structure and composition of gut microbiota were affected by multiple factors. In our study, the relatively higher gut microbiota diversity observed in TWA might be explained by environmental heterogeneity. Y and S were under the supervision of a professional shepherd and subsisted on high-quality grass pastures. In contrast, human interference forced TWA to travel longer distances to eat lower-quality indigenous plants in various habitats. Cao et al. (2009) reported that during the warm season, 16 plant species were edible for Y and S, while 19 plant species were edible for TWA. Differences in grazing strategy lead to differences in environmental heterogeneity. For wild animals, wide feeding ranges are beneficial for the diversification of food and gut microbial diversity. Conversely, domestic animals grazing under the control of herdsman are subject to reduced environment heterogeneity, which is not conducive for the development of microbial diversity. Thus, environment heterogeneity and food source diversity may be complementary. Therefore, we believed that diet and environmental heterogeneity significantly influence the differences between domestic and wild animals in terms of gut microbial diversity. The results of the present study were consistent with an earlier one indicating that among host behavior, geographical distance, environmental variation, and genetics, environmental variation was the principal predictor of host–microbiota associations (Song et al., 2020).

In this study, we systematically and comprehensively analyzed the differences in the gut microbiota of wild and domestic animals. Similar to previous studies (Liu et al., 2020b; Jiang et al., 2021), Bacteroidetes and Firmicutes, which were related to cellulose and hemicellulose decomposing, were found to be the most abundant bacterial phyla in our study. Moreover, indicator analysis showed these two phyla were represented by Y and TWA. The fact that Y possessed the potential to tolerate roughage through numerous Bacteroidetes consortiums has been reported (Guo et al., 2021). Here, the presence of Bacteroidetes indicator species in TWA suggests that these animals might be more tolerant to roughage than Y. This speculation is supported by the relatively higher herbage ADF and NDF digestibility and cellulose activity in the gut of TWA.

The SCFAs, the metabolites of forge fermentation by microorganisms, are important energy sources for the host, and they also maintain the homeostasis of the intestine (Popkes and Valenzano, 2020). Studies have shown that the main SCFAs in the gut are acetate and propionate (Fan et al., 2020). Consequently, we designated them as the main environmental factors and elucidated their relationships with microbial community indicator species. We found Actinobacteria were significantly positively correlated with propionate production in the gut of S while Planctomycetes was significantly positively correlated with the Shannon and Simpson indices. The foregoing bacteria did not predominate in the intestinal microflora. Nevertheless, they increased gut microbiota diversity and homogeneity and may have helped S to digest and absorb dietary nutrients. In TWA, all indicator species in the Bacteroidetes and Actinobacteria (but not Kiritimatiellaeota), as well as the genera *Prevotella_1* and *Rikenellaceae* promoted propionate production. For Y, however, only Firmicutes positively affected propionate production. As TWA had a diverse gut microbial consortium associated with propionic acid production, we speculated that this wild herbivore absorbed energy more effectively than the domestic herbivores (van Kessel et al., 2011). This advantage enabled TWA to cope with harsh environmental conditions and food scarcity.

Enterotypes were first discovered in the human gut microbiome and are roughly divided into *Bacteroidetes, Prevotella*, and *Ruminococcus*. These three taxa vary widely in terms of energy processing, vitamin biosynthesis and, by extension, impact host health (Arumugam et al., 2011). Guo et al. (2021) reported that yak shifted their enterotypes in response to dietary changes between the warm and cold seasons so that they could utilize N and energy most effectively. Fan et al. (2020) revealed the formation of various enterotypes helped QTP pika adapt to complex food conditions. The present study provided biological insight into gut enterotype clusters and functions as well as functional genomic information for wild and domestic herbivores. The *Akkermansia, Ruminococcaceae* sp, and *Bacteroides* enterotype which associated with high protein and low fiber forage nutrition belonged to Y and S, whereas, the *Prevotella_1, WCHB1-41*, and *Treponema 2* enterotype with low protein and high fiber belonging to TWA. It was suggested that the proportion of protein and carbohydrate contents mediated the host's enterotype shift, at least in wild baboons (Ren et al., 2016) and domestic yak (Guo et al., 2021). In our study, the variations in dietary protein and fiber contents could provide an attractive explanation for the differentiation of enterotype, and might also contribute to determine the enterotype for high-altitude herbivores on QTP. Y and S are indigenous livestock grouped into a single enterotype. This finding is an evidence for the existence of convergent gut microbe evolution (Xue et al., 2017). The formation of the TWA intestinal type might have been the result of long-term co-evolution between the host and the environment. It suggests that fixed enterotypes play key roles in animal adaptation to extreme environment. The present study also provided a mechanistic explanation as *Treponema 2, WCHB1-41*, and *Prevotella_1* participated in Arg and FA biosynthesis to utilize dietary N and generate energy for TWA subjected to harsh forage and environmental conditions. During the cold season on the QTP, domestic animals such as Y have lower N requirements and utilize dietary N more efficiently than cattle (Guo et al., 2012, 2021; Zhou et al., 2017). In the context of a growing overlap between the living space of wild and domestic animals, our study clarified the influences of *Treponema 2, WCHB1-41*, and *Prevotella_1* enterotypes on N and energy utilization in TWA and showed that these enterotypes play vital roles in mediating nutrient homeostasis in wild animals native to high altitude.

As 16S amplicon sequencing is limited (Qin et al., 2010), we elucidated gut microbiota structure and function via meta-genomic sequencing. The compositions of bacteria and archaea differed significantly among Y, S, and TWA. Bacteria were enriched in the TWA gut while archaea were enriched in the intestines of domestic animals. Certain grazing animals do not digest grass *per se*. Rather, they assimilate the bacteria decomposing the plant biomass into various nutrients (Niu et al., 2015). In the present study, we found that cellulolytic and proteolytic bacteria (Hastie et al., 2008; Liu et al., 2020a) such as *R. flavefaciens, P. ruminicola, B. fibrisolvens, R. albus, R. amylophilus*, and *F. succinogenes* were significantly more abundant in TWA than Y or S. These findings were consistent with the fact that TWA had relatively higher DM digestibility. Therefore, we concluded that the comparatively higher abundance of functional bacteria in the gut of TWA improves forage digestibility and produces high SCFAs concentrations to help the host efficiently use the forage, obtain energy, and adapt to environments with relatively less or inferior forage. When H_2_ accumulates during bacterial catabolism, archaeal growth is stimulated by H_2_ incorporation into greenhouse gases (GHG) such as methane and carbon dioxide (Matarazzo et al., 2012; Hoffmann et al., 2013). In the present study, *Methanobrevibacter, Methanosphaera*, and *Methanobacteriun* (Shi et al., 2014) associated with methane production were enriched in the intestinal tracts of S and Y. The methane emission from herbivorous not only results in environmental problems but also represents a significant source of energy loss to animals (Lan and Yang, 2019). A previous study revealed that Y and S were sustainable high-altitude mammals as their unique microbiome genes and structures could aid in the biological control of GHG emissions (Zhang et al., 2016). Here, we did not perform laboratory-level gas production assays on TWA, Y, or S from the perspective of the gut microbiome functional analysis; however, TWA might be more sustainable than Y or S as the former have lower methane emission potential than their domestic animal co-residents and might use energy more efficiently to cope with the perennial cold climate of the QTP.

The capability of herbivores to transform plant biomass into fermentable sugars depends entirely on the polysaccharide-hydrolyzing enzymes produced by intestinal microflora (Bohra et al., 2019). β-Glucosidase (EC 3.2.1.21) degrades plant cellulose by hydrolyzing the terminal non-reducing β-D-glucose bond (Xiros et al., 2013). Arabinanase (EC 3.2.1.99), xylanase (EC 3.2.1.8), and β-xylosidase (EC 3.2.1.37) synergistically degrade hemicellulose in plant cell walls (Sunna and Antranikian, 1997). The carbohydrate-active enzymes database (CAZy) provides information on CAZyme families including GHs, GTs, PLs, CEs, CBMs, and AAs. Of these, GHs are the most diverse and abundant (Cantarel et al., 2009). In the present study, we described the top 12 CAZyme families and enzymes. GH43, GH3, GH31, GH5, and GH10 decompose cellulose and hemicellulose and were more abundant in TWA than S or Y. Conversely, enzymes digesting starch and carbohydrate esters were comparatively more enriched in domestic animals. These discrepancies may be explained by the differences in the host feeding niches. TWA usually feeds on forage grass with high cellulose content whereas domestic animals selectively consume high-quality forage under the intervention of herdsmen. Convergent evolution of the functional genes of intestinal flora is also the consequence of host adaptation.

## Conclusion

The feeding niche heterogeneity is one of the main factors leading to the differences in gut microbiota among Y, S, and TWA. As the native herbivorous domestic livestock, the gut microbiota of Y and S exhibit convergent evolution characterized by the similar community and diversity and the same enterotype. Moreover, the intestinal flora of bath animals have an advantage in degrading plant starch and esterase. The indigenous TWA are highly adapted to the low temperatures, hypoxia, and high-fiber, low-protein native vegetation of this harsh environment in part because the composition, structure, and diversity of their gut microbiota are distinct from those of their domestic livestock co-residents. Furthermore, the intestinal microflora of TWA is characterized by high diversity, high nitrogen utilization efficiency and energy generation, abundant cellulolytic bacteria, high enzyme activity, and numerous gene families encoding glycoside hydrolases. The present study revealed the unique gut microbiota-mediated mechanisms by which TWA adapts to abiotic stress and poor forage quality. It also provides an important theoretical basis for the protection of wild animals indigenous to the QTP and the development and therapeutic application of intestinal microflora.

## Data Availability Statement

The raw sequence data reported in this paper have been deposited in Genome Sequence Archive (Wang et al., 2017) in BIG (BIG Data Center Members, 2019), Beijing Institute of Genomics (BIG), Chinese Academy of Sciences, under the accession numbers CRA006663615 (http://bigd.big.ac.cn/gsa).

## Ethics Statement

The animal study was reviewed and approved by Northwest Institute of Plateau Biology, CAS-Institutional Animal Care and Use Committee (OGRD# 2016YFC0501905).

## Author Contributions

XH, XZ, SX, LH, NZ, XW, CL, and YC contributed to sample collection and reviewed the manuscript. HL designed the research, analyzed the data, and drafted the manuscript. All authors have read and agreed to the final version of the manuscript.

## Funding

This work was supported by the National Natural Science Foundation of China (grant no. 32100100), Fundamental Research Project of Qinghai Province (2022-ZJ-943Q), National Key Research and Development Program of China (2021YFD1600200), and the Second Comprehensive Scientific Investigation of the Qinghai-Tibet Plateau (2019QZKK040104).

## Conflict of Interest

The authors declare that the research was conducted in the absence of any commercial or financial relationships that could be construed as a potential conflict of interest. The Reviewer XZ declared a shared affiliation with the authors HL, NZ, LH, XW, CL, XZ, and SX at the time of the review.

## Publisher's Note

All claims expressed in this article are solely those of the authors and do not necessarily represent those of their affiliated organizations, or those of the publisher, the editors and the reviewers. Any product that may be evaluated in this article, or claim that may be made by its manufacturer, is not guaranteed or endorsed by the publisher.

## References

[B1] AOAC (2000). Official Methods of Analysis of AOAC. 17th ed. Rockville, Maryland: AOAC International.

[B2] Arumugam M. Raes J. Pelletier E. Le Paslier D. Yamada T. Mende D. R.. (2011). Enterotypes of the human gut microbiome. Nature 473, 174–180. 10.1038/nature09944 21508958 PMC3728647

[B3] Bengtsson-Palme J. Hartmann M. Eriksson K. M. Pal C. Thorell K. Larsson D. G. J.. (2015). METAXA2: improved identification and taxonomic classification of small and large subunit rRNA in metagenomic data. Mol. Ecol. Resourc. 15, 1403–1414. 10.1111/1755-0998.12399 25732605

[B4] BIG Data Center Members (2019). Database resources of the BIG Data Center in 2019. Nucl. Acids Res. 47, D8–D14. 10.1093/nar/gky993 30365034 PMC6323991

[B5] Bohra V. Dafale N. A. Purohit H. J. (2019). Understanding the alteration in rumen microbiome and CAZymes profile with diet and host through comparative metagenomic approach. Archiv. Microbiol. 201, 1385–1397. 10.1007/s00203-019-01706-z 31338542

[B6] Boyd B. (2010). On the Origin of Stories: Evolution, Cognition, and Fiction. Cambridge, MA: Harvard University Press. 10.2307/j.ctvjf9xvk

[B7] Brune A. Ohkuma M. (2010). Role of the termite gut microbiota in symbiotic digestion, in Biology of Termites: A Modern Synthesis (Springer), 439–475. 10.1007/978-90-481-3977-4_16

[B8] Buchfink B. Xie C. Huson D. H. (2015). Fast and sensitive protein alignment using DIAMOND. Nat. Methods 12, 59–60. 10.1038/nmeth.3176 25402007

[B9] Cantarel B. L. Coutinho P. M. Rancurel C. Bernard T. Lombard V. Henrissat B. (2009). The Carbohydrate-Active EnZymes database (CAZy): an expert resource for glycogenomics. Nucl. Acids Res. 37, D233–D238. 10.1093/nar/gkn663 18838391 PMC2686590

[B10] Cao Y. Zhang T. Lian X. Cui Q. Deng D. Su J. (2009). Diet overlap among selected ungulates in Kekexili region, Qinghai province. Sichuan J. Zool. 28, 49–54. 10.1360/972009-1142

[B11] Caporaso J. G. Kuczynski J. Stombaugh J. Bittinger K. Bushman F. D. Costello E. K.. (2010). QIIME allows analysis of high-throughput community sequencing data. Nat. Methods 7, 335–336. 10.1038/nmeth.f.303 20383131 PMC3156573

[B12] Clarke K. R. (1993). Non-parametric multivariate analyses of changes in community structure. Austr. J. Ecol. 18, 117–143. 10.1111/j.1442-9993.1993.tb00438.x

[B13] Costea P. I. Hildebrand F. Arumugam M. Bäckhed F. Blaser M. J. Bushman F. D.. (2018). Enterotypes in the landscape of gut microbial community composition. Nat. Microbiol. 3, 8–16. 10.1038/s41564-017-0072-8 29255284 PMC5832044

[B14] Cui Z. Wu S. Li J. Yang Q.-E. Chai S. Wang L.. (2020). Effect of alfalfa hay and starter feeding intervention on gastrointestinal microbial community, growth and immune performance of yak calves. Front. Microbiol. 11:994. 10.3389/fmicb.2020.00994 32582049 PMC7287295

[B15] Dong Q. Zhao X. Ma Y. Xu S. Li Q. (2006). Live-weight gain, apparent digestibility, and economic benefits of yaks fed different diets during winter on the Tibetan plateau. Livestock Sci. 101, 199–207. 10.1016/j.livprodsci.2005.11.009

[B16] Edgar R. C. Haas B. J. Clemente J. C. Quince C. Knight R. (2011). UCHIME improves sensitivity and speed of chimera detection. Bioinformatics 27, 2194–2200. 10.1093/bioinformatics/btr381 21700674 PMC3150044

[B17] Fan C. Zhang L. Fu H. Liu C. Li W. Cheng Q.. (2020). Enterotypes of the gut microbial community and their response to plant secondary compounds in plateau pikas. Microorganisms 8:1311. 10.3390/microorganisms8091311 32872148 PMC7563992

[B18] Fan Q. Cui X. Wang Z. Chang S. Wanapat M. Yan T.. (2021). Rumen microbiota of Tibetan Sheep (*Ovis aries*) adaptation to extremely cold season on the Qinghai-Tibetan Plateau. Front. Vet. Sci. 8:554. 10.3389/fvets.2021.673822 34113677 PMC8185353

[B19] Fu H. Zhang L. Fan C. Liu C. Li W. Li J.. (2021). Domestication shapes the community structure and functional metagenomic content of the Yak fecal microbiota. Front. Microbiol. 12:594075. 10.3389/fmicb.2021.594075 33897627 PMC8059439

[B20] Gao H. Chi X. Qin W. Wang L. Song P. Cai Z.. (2019). Comparison of the gut microbiota composition between the wild and captive Tibetan wild ass (*Equus kiang*). J. Appl. Microbiol. 126, 1869–1878. 10.1111/jam.14240 30825354 PMC6849810

[B21] Gomez A. Sharma A. K. Grev A. Sheaffer C. Martinson K. (2021). The horse gut microbiome responds in a highly individualized manner to forage lignification. J. Equine Vet. Sci. 96:103306. 10.1016/j.jevs.2020.103306 33349409

[B22] Grieneisen L. E. Charpentier M. J. Alberts S. C. Blekhman R. Bradburd G. Tung J.. (2019). Genes, geology and germs: gut microbiota across a primate hybrid zone are explained by site soil properties, not host species. Proc. Royal Soc. B 286:20190431. 10.1098/rspb.2019.0431 31014219 PMC6501927

[B23] Guo N. Wu Q. Shi F. Niu J. Zhang T. Degen A. A.. (2021). Seasonal dynamics of diet–gut microbiota interaction in adaptation of yaks to life at high altitude. NPJ Biofilms Microbiomes 7, 1–11. 10.1038/s41522-021-00207-6 33879801 PMC8058075

[B24] Guo X. Zhang Y. Zhou J. Long R. Xin G. Qi B.. (2012). Nitrogen metabolism and recycling in yaks (*Bos grunniens*) offered a forage–concentrate diet differing in N concentration. Anim. Prod. Sci. 52, 287–296. 10.1071/AN11208

[B25] Hastie P. M. Mitchell K. Murray J.-A. M. (2008). Semi-quantitative analysis of *Ruminococcus flavefaciens, Fibrobacter succinogenes* and *Streptococcus bovis* in the equine large intestine using real-time polymerase chain reaction. Br. J. Nutr. 100, 561–568. 10.1017/S0007114508968227 18377691

[B26] Hildebrand F. Nguyen T. L. A. Brinkman B. Yunta R. G. Cauwe B. Vandenabeele P.. (2013). Inflammation-associated enterotypes, host genotype, cage and inter-individual effects drive gut microbiota variation in common laboratory mice. Genome Biol. 14, 1–15. 10.1186/gb-2013-14-1-r4 23347395 PMC4053703

[B27] Hintz H. Schryver H. Stevens C. (1978). Digestion and absorption in the hindgut of nonruminant herbivores. J. Anim. Sci. 46, 1803–1807. 10.2527/jas1978.4661803x 357371

[B28] Hoffmann C. Dollive S. Grunberg S. Chen J. Li H. Wu G. D.. (2013). Archaea and fungi of the human gut microbiome: correlations with diet and bacterial residents. PLoS ONE 8:e66019. 10.1371/journal.pone.0066019 23799070 PMC3684604

[B29] Jiang F. Gao H. Qin W. Song P. Wang H. Zhang J.. (2021). Marked seasonal variation in structure and function of gut microbiota in forest and alpine musk deer. Front. Microbiol. 12:699797. 10.3389/fmicb.2021.699797 34552569 PMC8450597

[B30] Karlsson F. H. Fåk F. Nookaew I. Tremaroli V. Fagerberg B. Petranovic D.. (2012). Symptomatic atherosclerosis is associated with an altered gut metagenome. Nat. Commun. 3, 1–8. 10.1038/ncomms2266 23212374 PMC3538954

[B31] Karlsson F. H. Tremaroli V. Nookaew I. Bergström G. Behre C. J. Fagerberg B.. (2013). Gut metagenome in European women with normal, impaired and diabetic glucose control. Nature 498, 99–103. 10.1038/nature12198 23719380

[B32] Kavanagh S. Lynch P. B. O'Mara F. Caffrey P. J. (2001). A comparison of total collection and marker technique for the measurement of apparent digestibility of diets for growing pigs. Anim. Feed Sci. Technol. 89, 49–58. 10.1016/S0377-8401(00)00237-6

[B33] Kumar V. Rawat J. Patil R. C. Barik C. R. Purohit S. Jaiswal H.. (2021). Exploring the functional significance of novel cellulolytic bacteria for the anaerobic digestion of rice straw. Renew. Energy 169, 485–497. 10.1016/j.renene.2021.01.002

[B34] Lan W. Yang C. (2019). Ruminal methane production: associated microorganisms and the potential of applying hydrogen-utilizing bacteria for mitigation. Sci. Tot. Environ. 654, 1270–1283. 10.1016/j.scitotenv.2018.11.180 30841400

[B35] Ley R. E. Hamady M. Lozupone C. Turnbaugh P. J. Ramey R. R. Bircher J. S.. (2008). Evolution of mammals and their gut microbes. Science 320, 1647–1651. 10.1126/science.1155725 18497261 PMC2649005

[B36] Li H. Li T. Berasategui A. Rui J. Zhang X. Li C.. (2017). Gut region influences the diversity and interactions of bacterial communities in pikas (*Ochotona curzoniae* and *Ochotona daurica*). FEMS Microbiol. Ecol. 93:fix149. 10.1093/femsec/fix149 29106508

[B37] Li W. Godzik A. (2006). Cd-hit: a fast program for clustering and comparing large sets of protein or nucleotide sequences. Bioinformatics 22, 1658–1659. 10.1093/bioinformatics/btl158 16731699

[B38] Liu H. Hu L. Han X. Zhao N. Xu T. Ma L.. (2020a). Tibetan sheep adapt to plant phenology in alpine meadows by changing rumen microbial community structure and function. Front. Microbiol. 2547:587558. 10.3389/fmicb.2020.587558 33193243 PMC7649133

[B39] Liu H. Xu T. Xu S. Ma L. Han X. Wang X.. (2019). Effect of dietary concentrate to forage ratio on growth performance, rumen fermentation and bacterial diversity of Tibetan sheep under barn feeding on the Qinghai-Tibetan plateau. PeerJ 7:e7462. 10.7717/peerj.7462 31404417 PMC6686838

[B40] Liu H. Zhao X. Han X. Xu S. Zhao L. Hu L.. (2020b). Comparative study of gut microbiota in Tibetan wild asses (*Equus kiang*) and domestic donkeys (*Equus asinus*) on the Qinghai-Tibet plateau. PeerJ 8:e9032. 10.7717/peerj.9032 32547852 PMC7276150

[B41] Liu W. Wang Q. Song J. Xin J. Zhang S. Lei Y.. (2021). Comparison of gut microbiota of yaks from different geographical regions. Front. Microbiol. 12:666940. 10.3389/fmicb.2021.666940 34163445 PMC8216380

[B42] Liu X. Sha Y. Dingkao R. Zhang W. Lv W. Wei H.. (2020c). Interactions between rumen microbes, VFAs, and host genes regulate nutrient absorption and epithelial barrier function during cold season nutritional stress in Tibetan sheep. Front. Microbiol. 2802:593062. 10.3389/fmicb.2020.593062 33250882 PMC7674685

[B43] Liu Y. Luo J. Dou J. Yan B. Ren Q. Tang B.. (2020d). The sequence and de novo assembly of the wild yak genome. Sci. Data 7, 1–8. 10.1038/s41597-020-0400-3 32094352 PMC7039982

[B44] Luo R. Liu B. Xie Y. Li Z. Huang W. Yuan J.. (2012). SOAPdenovo2: an empirically improved memory-efficient short-read de novo assembler. Gigascience 1:2047–2217X-2041-2018. 10.1186/2047-217X-1-18 23587118 PMC3626529

[B45] Ma L. Xu S. Liu H. Xu T. Hu L. Zhao N.. (2019a). Yak rumen microbial diversity at different forage growth stages of an alpine meadow on the Qinghai-Tibet Plateau. PeerJ 7:e7645. 10.7717/peerj.7645 31579584 PMC6754979

[B46] Ma Y. Ma S. Chang L. Wang H. Ga Q. Ma L.. (2019b). Gut microbiota adaptation to high altitude in indigenous animals. Biochem. Biophys. Res. Commun. 516, 120–126. 10.1016/j.bbrc.2019.05.085 31196622

[B47] Martinson V. G. Douglas A. E. Jaenike J. (2017). Community structure of the gut microbiota in sympatric species of wild Drosophila. Ecol. Lett. 20, 629–639. 10.1111/ele.12761 28371064

[B48] Masella A. P. Bartram A. K. Truszkowski J. M. Brown D. G. Neufeld J. D. (2012). PANDAseq: paired-end assembler for illumina sequences. BMC Bioinformati. 13, 1–7. 10.1186/1471-2105-13-31 22333067 PMC3471323

[B49] Matarazzo F. Ribeiro A. Faveri M. Taddei C. Martinez M. Mayer M. (2012). The domain Archaea in human mucosal surfaces. Clin. Microbiol. Infect. 18, 834–840. 10.1111/j.1469-0691.2012.03958.x 22827611

[B50] Miller G. L. (1959). Use of dinitrosalicylic acid reagent for determination of reducing sugar. Analyt. Chem. 31, 426–428. 10.1021/ac60147a03033274222

[B51] Nielsen H. B. Almeida M. Juncker A. S. Rasmussen S. Li J. Sunagawa S.. (2014). Identification and assembly of genomes and genetic elements in complex metagenomic samples without using reference genomes. Nat. Biotechnol. 32, 822–828. 10.1038/nbt.2939 24997787

[B52] Niu Q. Li P. Hao S. Zhang Y. Kim S. W. Li H.. (2015). Dynamic distribution of the gut microbiota and the relationship with apparent crude fiber digestibility and growth stages in pigs. Sci. Rep. 5, 1–7. 10.1038/srep09938 25898122 PMC4404679

[B53] Oh J. Byrd A. L. Deming C. Conlan S. Kong H. H. Segre J. A. (2014). Biogeography and individuality shape function in the human skin metagenome. Nature 514, 59–64. 10.1038/nature13786 25279917 PMC4185404

[B54] Oksanen J. Blanchet F. G. Friendly M. Kindt R. Legendre P. McGlinn D.. (2013). Community Ecology Package. R package version 2.5–7. Available online at: https://CRAN.R-project.org/package=vegan

[B55] Popkes M. Valenzano D. R. (2020). Microbiota–host interactions shape ageing dynamics. Philos. Trans. Royal Soc. B 375:20190596. 10.1098/rstb.2019.0596 32772667 PMC7435156

[B56] Qiao H. Wang X. Wang W. Luo Z. Tang K. Huang Y.. (2018). From nature reserve to national park system pilot: changes of environmental coverage in the Three-River-Source National Park and implications for amphibian and reptile conservation. Biodiv. Sci. 26:202. 10.17520/biods.2017305

[B57] Qin J. Li R. Raes J. Arumugam M. Burgdorf K. S. Manichanh C.. (2010). A human gut microbial gene catalogue established by metagenomic sequencing. Nature 464, 59–65. 10.1038/nature08821 20203603 PMC3779803

[B58] Qu Y. Chen C. Xiong Y. She H. Zhang Y. E. Cheng Y.. (2020). Rapid phenotypic evolution with shallow genomic differentiation during early stages of high elevation adaptation in Eurasian Tree Sparrows. Nat. Sci. Rev. 7, 113–127. 10.1093/nsr/nwz138 34692022 PMC8289047

[B59] Ren T. Grieneisen L. E. Alberts S. C. Archie E. A. Wu M. (2016). Development, diet and dynamism: longitudinal and cross-sectional predictors of gut microbial communities in wild baboons. Environ. Microbiol. 18, 1312–1325. 10.1111/1462-2920.12852 25818066 PMC5941927

[B60] Schaller G. B. (2000). Wildlife of the Tibetan Steppe. Chicago, IL: University of Chicago Press.

[B61] Scher J. U. Sczesnak A. Longman R. S. Segata N. Ubeda C. Bielski C.. (2013). Expansion of intestinal *Prevotella copri* correlates with enhanced susceptibility to arthritis. Elife 2:e01202. 10.7554/eLife.01202 24192039 PMC3816614

[B62] Shi W. Moon C. D. Leahy S. C. Kang D. Froula J. Kittelmann S.. (2014). Methane yield phenotypes linked to differential gene expression in the sheep rumen microbiome. Genome Res. 24, 1517–1525. 10.1101/gr.168245.113 24907284 PMC4158751

[B63] Shreiner A. B. Kao J. Y. Young V. B. (2015). The gut microbiome in health and in disease. Curr. Opin. Gastroenterol. 31:69. 10.1097/MOG.0000000000000139 25394236 PMC4290017

[B64] Song P. Qin W. Huang Y. Wang L. Cai Z. Zhang T. (2020). Grazing management influences gut microbial diversity of livestock in the same area. Sustainability 12:4160. 10.3390/su12104160

[B65] Sunna A. Antranikian G. (1997). Xylanolytic enzymes from fungi and bacteria. Crit. Rev. Biotechnol. 17, 39–67. 10.3109/07388559709146606 9118232

[B66] van Kessel M. A. Dutilh B. E. Neveling K. Kwint M. P. Veltman J. A. Flik G.. (2011). Pyrosequencing of 16S rRNA gene amplicons to study the microbiota in the gastrointestinal tract of carp (*Cyprinus carpio* L.). AMB Expr. 1, 1–9. 10.1186/2191-0855-1-41 22093413 PMC3226434

[B67] Van Soest P. v. Robertson J. Lewis B. (1991). Methods for dietary fiber, neutral detergent fiber, and nonstarch polysaccharides in relation to animal nutrition. J. Dairy Sci. 74, 3583–3597. 10.3168/jds.S0022-0302(91)78551-2 1660498

[B68] Vanessa L. Chia L. Niu K. Yang Y. Rob K. Zhang Q.. (2017). Diet vs. phylogeny: a comparison of gut microbiota in captive colobine monkey species. Microb. Ecol. 75, 515–527. 10.1007/s00248-017-1041-8 28735426

[B69] Wang Y. Song F. Zhu J. Zhang S. Yang Y. Chen T.. (2017). GSA: genome sequence archive. Genom. Proteom. Bioinformat. 15, 14–18. 10.1016/j.gpb.2017.01.001 28387199 PMC5339404

[B70] Xiros C. Topakas E. Christakopoulos P. (2013). Hydrolysis and fermentation for cellulosic ethanol production. Wiley Interdiscipl. Rev. 2, 633–654. 10.1002/wene.4920419480

[B71] Xue D. Chen H. Zhao X. Xu S. Hu L. Xu T.. (2017). Rumen prokaryotic communities of ruminants under different feeding paradigms on the Qinghai-Tibetan Plateau. Systemat. Appl. Microbiol. 40, 227–236. 10.1016/j.syapm.2017.03.006 28495244

[B72] Zhang H. Sparks J. B. Karyala S. V. Settlage R. Luo X. M. (2015). Host adaptive immunity alters gut microbiota. ISME J. 9, 770–781. 10.1038/ismej.2014.165 25216087 PMC4331585

[B73] Zhang Z. Xu D. Wang L. Hao J. Wang J. Zhou X.. (2016). Convergent evolution of rumen microbiomes in high-altitude mammals. Curr. Biol. 26, 1873–1879. 10.1016/j.cub.2016.05.012 27321997

[B74] Zhao L. Wang G. Siegel P. He C. Meng H. (2013). Quantitative genetic background of the host influences gut microbiomes in chickens. Sci. Rep. 3:srep01163. 10.1038/srep01163 23362462 PMC3557447

[B75] Zhao W. Wang Y. Liu S. Huang J. Zhai Z. He C.. (2015). The dynamic distribution of porcine microbiota across different ages and gastrointestinal tract segments. PLoS ONE 10:e0117441. 10.1371/journal.pone.0117441 25688558 PMC4331431

[B76] Zhao X. Xu T. Ellis J. He F. Hu L. Li Q. (2020). Rewilding the wildlife in Sangjiangyuan National Park, Qinghai-Tibetan Plateau. Ecosyst. Health Sustainability 6:1776643. 10.1080/20964129.2020.1776643

[B77] Zheng X. Qiu Y. Zhong W. Baxter S. Su M. Li Q.. (2013). A targeted metabolomic protocol for short-chain fatty acids and branched-chain amino acids. Metabolomics 9, 818–827. 10.1007/s11306-013-0500-6 23997757 PMC3756605

[B78] Zhou J. Zhong C. Liu H. Degen A. Titgemeyer E. Ding L.. (2017). Comparison of nitrogen utilization and urea kinetics between yaks (*Bos grunniens*) and indigenous cattle (*Bos taurus*). J. Anim. Sci. 95, 4600–4612. 10.2527/jas2017.1428 29108052

