# Supplementary material for: The Gut Microbiota Determines the High-Altitude Adaptability of Tibetan Wild Asses (Equus kiang) in Qinghai-Tibet Plateau
Source: Front Microbiol. 2022 Jul 18;13:949002. doi: 10.3389/fmicb.2022.949002 (PMC9342865; doi:10.3389/fmicb.2022.949002)
Supplement: Supplementary file 1 [file Table_1.docx]

**Supplementary Figure 1** Enterotype clusters identified by partitioning around method (PAM) based on the Bray-Curits dissimilarity (BC dissimilarity) and Jensen-Shannon distance (JSD). Clustering of gut microbiota taxa into enterotypes are presented in main text **Figure.5**. Principal coordinated analysis plots represent the two clusters of hosts gut microbiota identified by PAM based on the BC (**A**) and JSD (D) among relative abundance at the genus level (27 samples). Numbered white rectangles denote the centroid of each cluster and solid lines denote the distance of each sample from the centroid of enterotype cluster. Boxplots represent of the similarity between enterotype identified from BC and JSD from different enterotypes. Optimal number of clusters for PAM analysis based on BC (**C**) and (**F**) JSD among the relative abundance distributions at the genus level. Boxplot center values represent the median, and whiskers represented 0.75 times the interquartile range. Analysis of similarities (ANOSIM) was used for statistical testing between enterotype similarities. *P*-value, * <0.05, **<0.01, *** <0.001


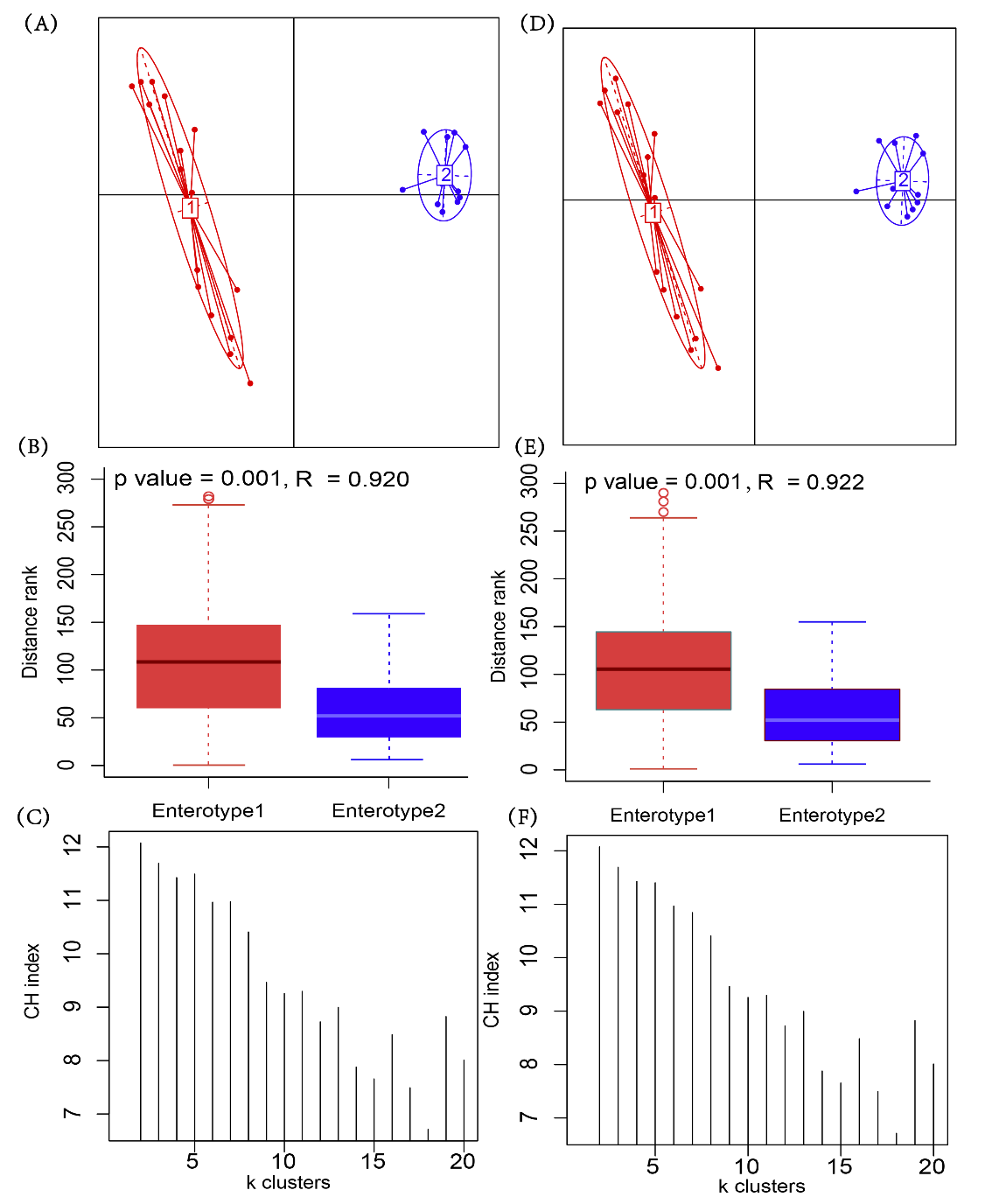


**Supplementary Figure 2** Lollipop charts showing indicator species of gut microbiota at phylum (**A**) and genus (**B**) level. Lollipop are colored by different microbiota at the phylum at genus level. A high value indicates a species has a high indicator power for corresponding group.


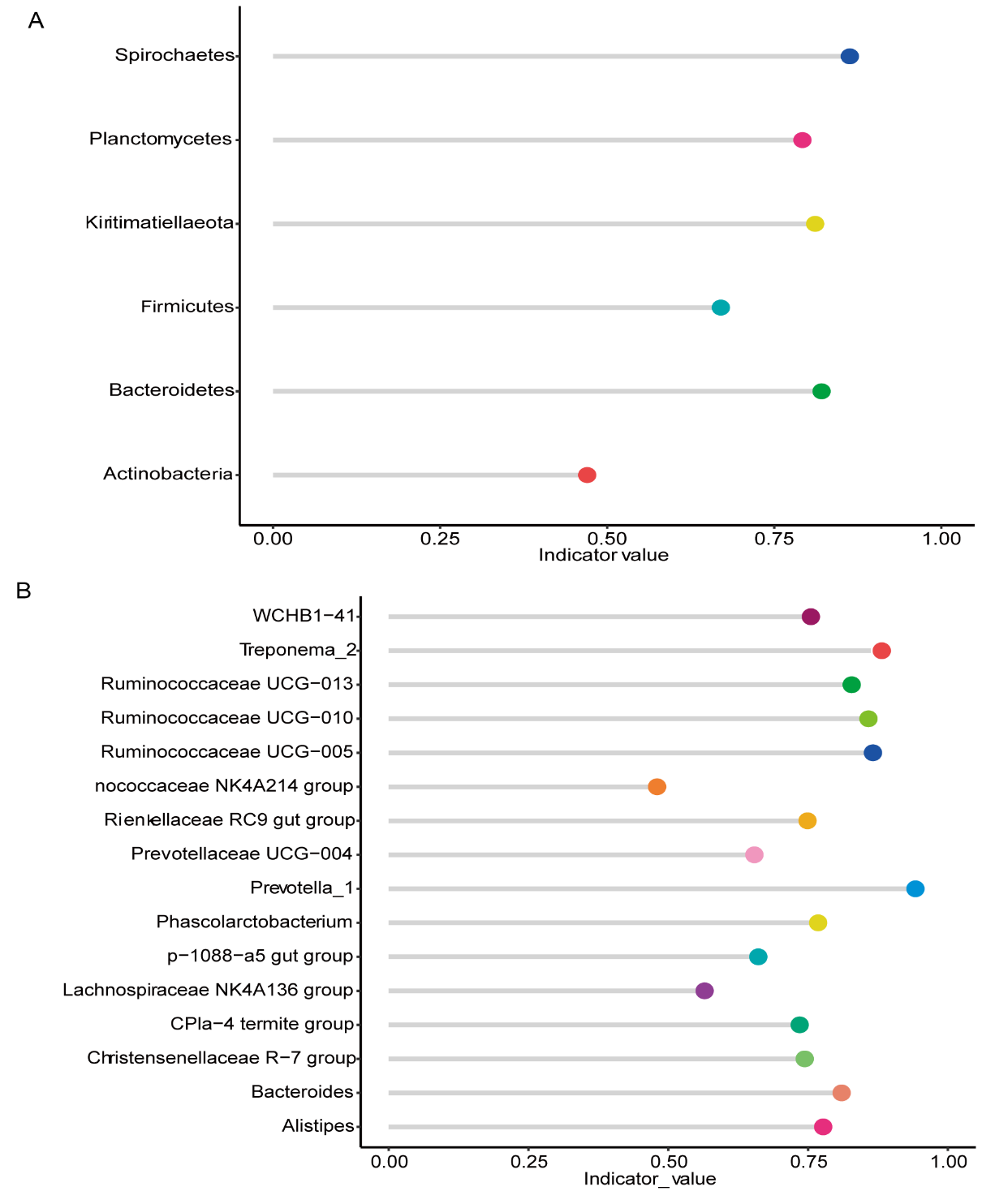


**Supplementary Figure 3** Relative abundances of the rest of four main contributors at the gut microbiota genus level taxa. All boxplot distributions are tested by non-parametric Kruskal-Wallis and Wilcoxon with FDR (false discoveryrate) correct *P* value and center values indicate the median and error bars. *** correct *P* < 0.05.


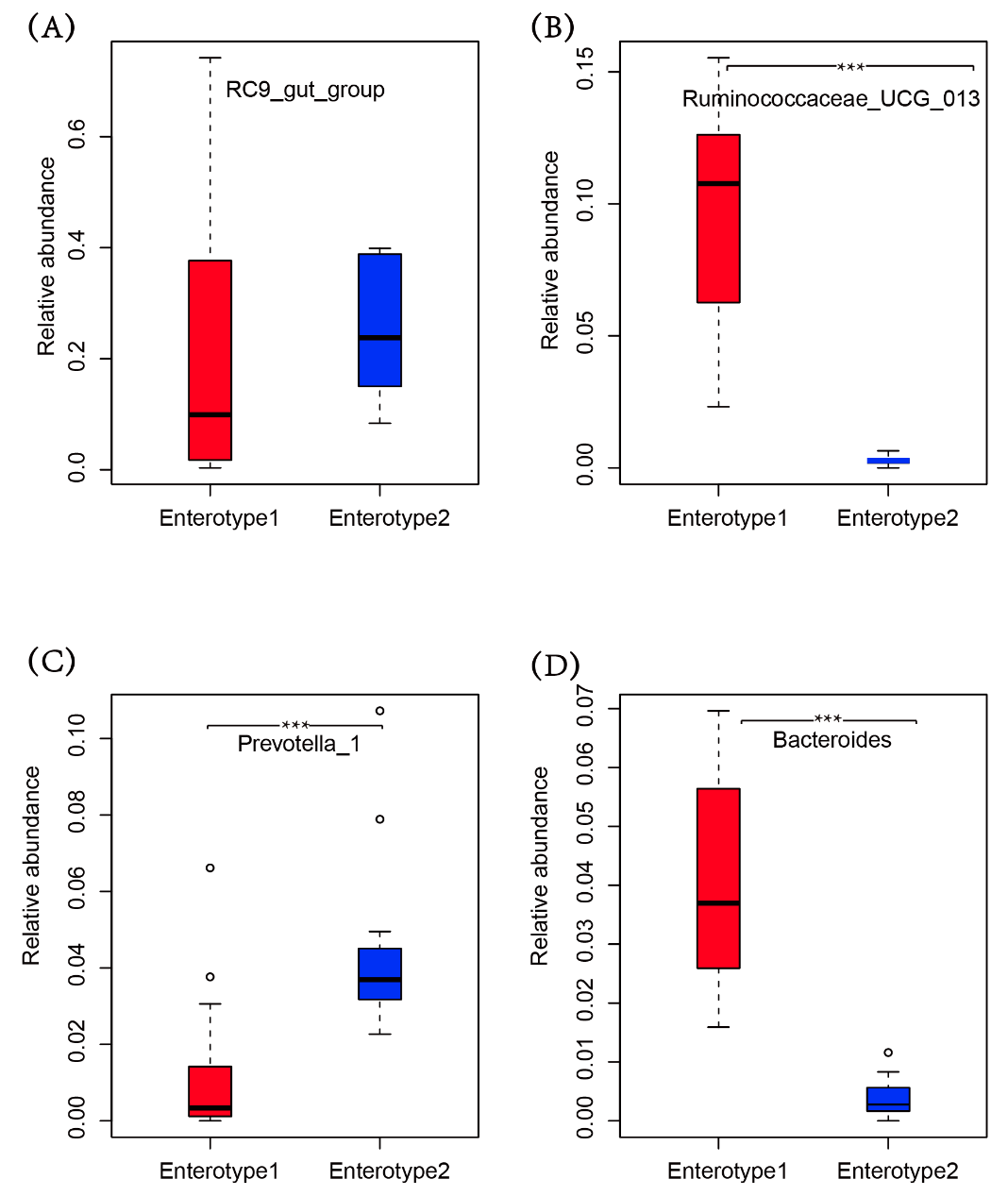


**Supplementary Figure 4** Relative abundances of top12 EC numbers. *correct *P* < 0.05*, ***correct *P* < 0.01*.*


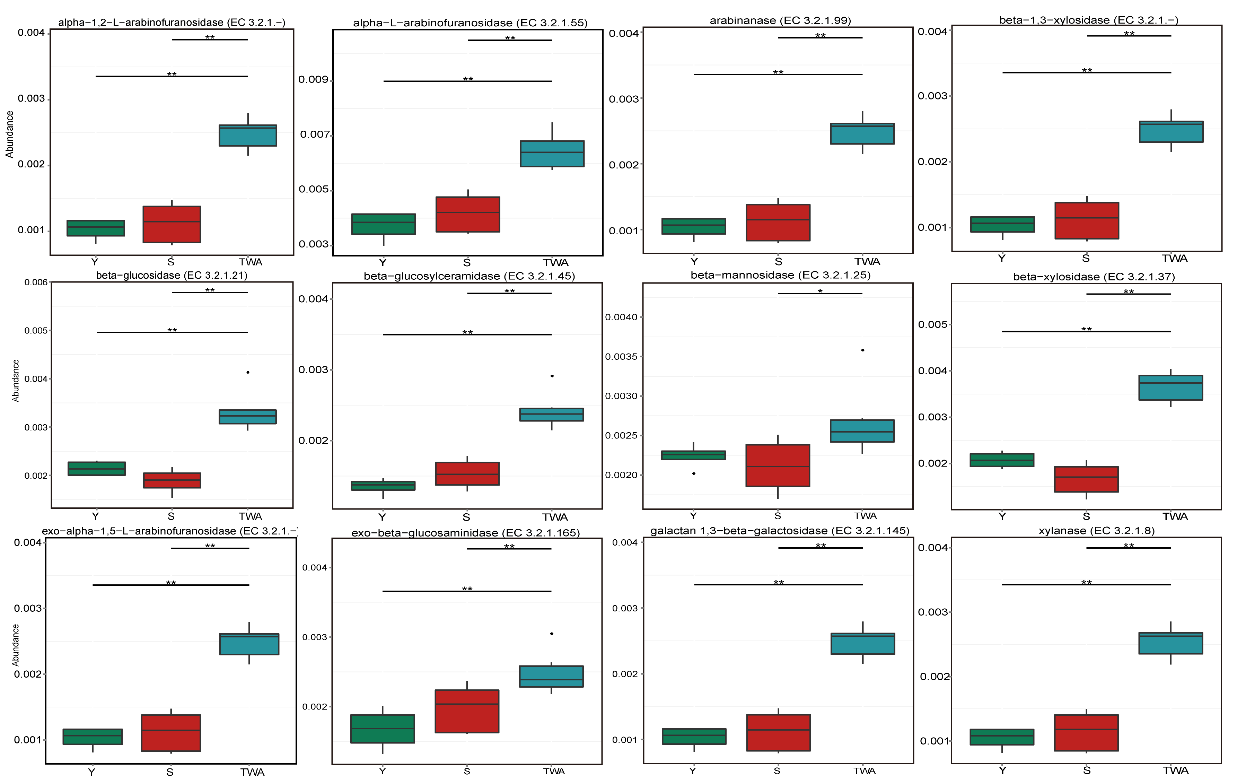


**Supplemental Table S1** The common forage nutrition contents among Y, S and TWA groups.

| **Nutrient content (%)** | **Groups^a^** | | | ***P*-values** |
| --- | --- | --- | --- | --- |
|  | **S** | **Y** | **TWA** |  |
| DM | 94.43±0.32^b^ | | 96.13±0.41^a^ | ＜0.05 |
| CP | 12.15±0.96^a^ | | 9.69±0.63^b^ | ＜0.05 |
| EE | 1.41±0.13 | | 1.13±0.06 | 0.06 |
| ADF | 22.49±1.45^b^ | | 27.17±2.07^a^ | ＜0.05 |
| NDF | 45.47±3.01^a^ | | 51.65±3.46^b^ | ＜0.05 |
| Ash | 15.23±1.28^a^ | | 13.25±1.06^b^ | ＜0.06 |
| NFC | 28.15±2.23^a^ | | 24.41±2.49^b^ | ＜0.07 |
| Dominant species of herbages | *Kobresia tibetica*, *Kobresia pygmaea* | | *Stipa purpurea, Carex moorcrofiii* |  |

^a^ S, Tibetan sheep; Y, Yak; TWA, Tibetan wild asses. In the same row, values with different letters mean significant different (*P* < 0.05), while with the same letter or no superscripts mean no significant difference (*P* > 0.05). The same as bellow. As yak and Tibetan sheep are raised in mixed groups, the forage they eat is considered to be the same.

**Supplementary Table S2** The sequence information of 16S rRNA amplicons.

| **Sample Name** | **Total tags** | **Clean tags** | **Singleton** | **Effective tags** | **Effective Ratio (%)** | **OTUs** |
| --- | --- | --- | --- | --- | --- | --- |
| TWA.1 | 83715 | 73203 | 10512 | 62691 | 85.64 | 1415 |
| TWA.2 | 82480 | 69213 | 13267 | 55946 | 80.83 | 1272 |
| TWA.3 | 96125 | 83968 | 12157 | 71811 | 85.52 | 1472 |
| TWA.4 | 86410 | 72156 | 14254 | 57902 | 80.25 | 1094 |
| TWA.5 | 93771 | 82046 | 11725 | 70321 | 85.71 | 1228 |
| TWA.6 | 97269 | 83478 | 13791 | 69687 | 83.48 | 1264 |
| TWA.7 | 90727 | 82877 | 7850 | 75027 | 90.53 | 1000 |
| TWA.8 | 108120 | 84928 | 23192 | 61736 | 72.69 | 1033 |
| TWA.9 | 96964 | 89632 | 7332 | 82300 | 91.82 | 858 |
| TWA.10 | 92091 | 82955 | 9136 | 73819 | 88.99 | 1148 |
| TWA.11 | 104952 | 94203 | 10749 | 83454 | 88.59 | 1209 |
| S.1 | 71614 | 62892 | 8722 | 54170 | 86.13 | 1111 |
| S.2 | 62399 | 55935 | 6464 | 49471 | 88.44 | 979 |
| S.3 | 67912 | 62365 | 5547 | 56818 | 91.11 | 800 |
| S.4 | 79361 | 65072 | 14289 | 50783 | 78.04 | 865 |
| S.5 | 70865 | 62817 | 8048 | 54769 | 87.19 | 961 |
| S.6 | 87004 | 73667 | 13337 | 60330 | 81.90 | 723 |
| S.7 | 74053 | 67206 | 6847 | 60359 | 89.81 | 936 |
| Y.1 | 93129 | 85805 | 7324 | 78481 | 91.46 | 756 |
| Y.2 | 98397 | 90172 | 8225 | 81947 | 90.88 | 1053 |
| Y.3 | 81715 | 74828 | 6887 | 67941 | 90.80 | 895 |
| Y.4 | 109744 | 86345 | 23399 | 62946 | 72.90 | 1306 |
| Y.5 | 165986 | 144217 | 21769 | 122448 | 84.91 | 1434 |
| Y.6 | 116286 | 97321 | 18965 | 78356 | 80.51 | 1328 |
| Y.7 | 89271 | 76805 | 12466 | 64339 | 83.77 | 1108 |
| Y.8 | 90074 | 80273 | 9801 | 70472 | 87.79 | 1113 |
| Y.9 | 107141 | 93854 | 13287 | 80567 | 85.84 | 1270 |
| **Total number** | * | **2178233** | * | **1858891** | * | **29631** |
| **Average number** | 93305.1 | **80675.3** | * | **68847.8** | **85.39** | **1097.44** |

**Supplementary Table S3** The relative abundances of gut microbiota from each host at the phylum level.

| **Phylum** | **TWA** | **Y** | **S** | **Average (%)** |
| --- | --- | --- | --- | --- |
| *Firmicutes* | 38.27 | 56.36 | 38.03 | 44.22 |
| *Bacteroidetes* | 28.31 | 22.15 | 11.35 | 20.60 |
| *Planctomycetes* | 11.35 | 1.69 | 32.26 | 15.10 |
| *Kiritimatiellaeota* | 4.77 | 0.35 | 0.05 | 1.72 |
| *Verrucomicrobia* | 3.88 | 14.56 | 9.68 | 9.37 |
| *Spirochaetes* | 4.23 | 0.04 | 0.68 | 1.65 |
| *Proteobacteria* | 2.06 | 2.08 | 1.37 | 1.84 |
| *Euryarchaeota* | 0.10 | 0.19 | 1.01 | 0.43 |
| *Actinobacteria* | 1.53 | 0.69 | 1.19 | 1.13 |
| *Synergistetes* | 1.50 | 0.00 | 0.00 | 0.50 |
| *Cyanobacteria* | 1.35 | 0.46 | 1.19 | 1.00 |
| *Epsilonbacteraeota* | 0.32 | 0.01 | 0.05 | 0.13 |
| *Acidobacteria* | 0.10 | 0.12 | 0.91 | 0.38 |
| *Gemmatimonadetes* | 0.06 | 0.14 | 0.33 | 0.18 |
| *Fibrobacteres* | 0.51 | 0.01 | 0.01 |  |
| *Patescibacteria* | 0.10 | 0.59 | 0.80 | 0.50 |
| *Chloroflexi* | 0.07 | 0.01 | 0.48 | 0.19 |
| *Tenericutes* | 0.06 | 0.15 | 0.08 | 0.10 |
| *Lentisphaerae* | 0.06 | 0.04 | 0.02 | 0.04 |
| *Elusimicrobia* | 0.05 | 0.03 | 0.04 | 0.04 |

**Supplementary Table S4** The relative abundance of gut microbiota from each hosts at the phylum level.

| **Genus** | **TWA** | **Yak** | **Tibetan sheep** | **Average (%)** |
| --- | --- | --- | --- | --- |
| *p-1088-a5 gut group* | 10.77 | 1.40 | 22.66 | 11.61 |
| *Akkermansia* | 4.47 | 9.18 | 9.90 | 7.85 |
| *Ruminococcaceae UCG-005* | 0.95 | 16.15 | 5.43 | 7.51 |
| *Rikenellaceae RC9 gut group* | 2.99 | 6.15 | 2.83 | 3.99 |
| *WCHB1-41* | 1.66 | 5.76 | 5.23 | 4.22 |
| *Ruminococcaceae UCG-010* | 1.19 | 7.99 | 2.59 | 3.92 |
| *Phascolarctobacterium* | 6.89 | 1.83 | 0.62 | 3.11 |
| *Ruminococcaceae UCG-013* | 0.11 | 3.85 | 4.19 | 2.72 |
| *CPla-4 termite group* | 0.00 | 0.12 | 8.42 | 2.85 |
| *Christensenellaceae R-7 group* | 1.40 | 2.05 | 3.53 | 2.33 |
| *Ruminococcaceae NK4A214 group* | 2.47 | 1.50 | 2.08 | 2.02 |
| *Prevotellaceae UCG-004* | 1.06 | 3.37 | 1.71 | 2.04 |
| *Treponema 2* | 4.20 | 0.04 | 0.70 | 1.65 |
| *Lachnospiraceae NK4A136 group* | 2.15 | 1.38 | 1.03 | 1.52 |
| *Prevotella_1* | 3.27 | 0.29 | 0.17 | 1.24 |
| *Roseburia* | 1.47 | 1.25 | 0.79 | 1.17 |
| *Escherichia-Shigella* | 0.02 | 3.54 | 0.01 | 1.19 |
| *Bacteroides* | 0.15 | 1.95 | 1.36 | 1.15 |
| *Alistipes* | 0.15 | 2.00 | 1.22 | 1.12 |

**Supplementary Table S5** The basic information of metagenomic sequencing.

| **Sample ID** | **Raw Data (Mbp)** | **Raw reads** | **Clean Data (Mbp)** | **Clean_Q20** | **Clean_Q30** | **Clean GC(%)** | **Effective (%)** |
| --- | --- | --- | --- | --- | --- | --- | --- |
| TWA.1 | 13346 | 88974026 | 13337 | 96.86 | 91.41 | 46.51 | 99.935 |
| TWA.2 | 12787 | 85245942 | 12702 | 97.09 | 91.89 | 45.26 | 99.333 |
| TWA.3 | 12993 | 86617762 | 12934 | 97.06 | 91.81 | 44.84 | 99.547 |
| TWA.4 | 12156 | 81038790 | 12131 | 97.03 | 91.78 | 47.19 | 99.797 |
| TWA.5 | 12850 | 85668946 | 12830 | 97.16 | 92.03 | 48.39 | 99.845 |
| TWA.6 | 12501 | 83339564 | 12443 | 96.94 | 91.57 | 45.11 | 99.537 |
| TWA.7 | 13083 | 87219700 | 13073 | 97.07 | 91.81 | 44.92 | 99.922 |
| Y.1 | 12698 | 84650070 | 12611 | 96.97 | 91.68 | 45.62 | 99.322 |
| Y.2 | 12884 | 85895136 | 12839 | 96.82 | 91.34 | 47.16 | 99.646 |
| Y.3 | 13108 | 87388340 | 12996 | 97.23 | 92.22 | 43.65 | 99.145 |
| Y.4 | 13397 | 89311144 | 13313 | 96.97 | 91.63 | 46.4 | 99.378 |
| S.1 | 12376 | 82504456 | 12354 | 97.04 | 91.8 | 43.48 | 99.827 |
| S.2 | 12566 | 83775348 | 12551 | 97.03 | 91.82 | 45.75 | 99.879 |
| S.3 | 12716 | 84772206 | 12702 | 96.49 | 90.65 | 44.75 | 99.89 |
| S.4 | 12157 | 81049840 | 12137 | 97.06 | 91.84 | 43.74 | 99.832 |
| S.5 | 12241 | 81604362 | 12209 | 96.82 | 91.3 | 46.62 | 99.742 |

**Supplementary Table S6** The taxnomic and functional annotate information of metagenomic sequencing

| Total ORFs | 6,319,617 |
| --- | --- |
| Average ORFs | 371,742 |
| Gene catalogue | **3,766,521** |
| Complete ORFs | 1,378,793(36.61%) |
| Total length (Mbp) | 2,459.08 |
| Average length (bp) | **652.88** |
| GC percent | 45.17% |
| **Taxonomic Annotation** |  |
| Gene catalogue | 3,766,521 |
| Annotated on NR | **2,942,685(78.13%)** |
| Annotated on Unclassified | 19.25% |
| Annotated on Kingdom level | **80.75%** |
| Annotated on Phylum level | **75.38%** |
| Annotated on Class level | 67.93% |
| Annotated on Order level | 67.12% |
| Annotated on Family level | 53.47% |
| Annotated on Genus level | **49.38%** |
| Annotated on Species level | **34.31%** |
| **Functional Annotation** |  |
| Gene catalogue | 3,766,521 |
| Annotated on KEGG | 2,120,400(56.30%) |
| Annotated on KO | 1,256,293(33.35%)/4,549 |
| Annotated on EC | 768,411(20.40%)/1,736 |
| Annotated on pathway | 732,578(19.45%)/311 |
| Annotated on eggNOG | 2,101,283(55.79%) |
| Annotated on OG | 2,101,283(55.79%)/19,653 |
| Annotated on CAZy | **143,944(3.82%)** |
| CARD Annotation |  |
| Gene catalogue | 3766521 |
| Annotated on CARD | 961 |
| Annotated AROs | 526 |

**Supplementary Table S7** The concentration of cellulose decomposition and methan producing microbiota among the different hosts.

| Items | Y | S | TWA | *P*-value |
| --- | --- | --- | --- | --- |
| Genus level |  |  |  |  |
| *Methanobrevibacter* | 45791±1412^a^ | 26941±4551^a^ | 3395±551^b^ | 0.001 |
| *Methanosphaera* | 7168±300^a^ | 52110±745a | 13±2^b^ | 0.003 |
| *Methanobacterium* | 281±14^a^ | 204±30^a^ | 35±4^b^ | 0.003 |
| *Methanocorpusculum* | 27±1^b^ | 37±2^b^ | 227±22^a^ | 0.001 |
| *Methanosarcina* | 52±4 | 74±2 | 71±7 | 0.104 |
| *Methanomassiliicoccus* | 20±1^b^ | 18±1^b^ | 35±2^a^ | 0.001 |
| Species level |  |  |  |  |
| *Fibrobacter succinogenes* | 13±1^a^ | 7±1^b^ | 68±5^a^ | 0.001 |
| *Ruminococcus flavefaciens* | 1568±130^b^ | 3281.2±111^b^ | 10517±427^a^ | 0.001 |
| *Ruminococcus albus* | 579±319^b^ | 827±10^a^ | 1552±96^a^ | 0.001 |
| *Butyrivibrio fibrisolvens* | 493±87^b^ | 429±28^b^ | 1047±68^a^ | 0.001 |
| *Ruminobacter amylophilus* | 8±0.35^b^ | 6±0.29^b^ | 21±1.41^a^ | 0.001 |
| *Prevotella ruminicola* | 488±26^b^ | 302±20^b^ | 2753±158^a^ | 0.001 |

**Supplementary Table S8** Definiation of cellulase, hemicellulase, starch, and esterase based on their catalytic domains. This table was curated based on the CAZymes datatabase (http://www.cazy.org/ accessed on 3 December, 2021).

| **Enzyme Class** | **Enzyme Name** | **Glycoside Hydrolase (GH)** | **Polysaccharide Lyase (PL)** | **Carbohydrate Esterase (CE)** | **GlycosylTransferase (GT)** |
| --- | --- | --- | --- | --- | --- |
| ***Cellulase*** |  |  |  |  |  |
| EC:2.4.1.12 | cellulose synthase |  |  |  | 2 |
| EC:3.2.1.37 | 1,4-β-xylosidase | 3 |  |  |  |
| EC:3.2.1.20 | α-glucosidase | 31 |  |  |  |
| EC:3.2.1.4 | endo-β-1,4-glucanase | 5 |  |  |  |
| ***Hemicellulase*** | |  |  |  |  |
| EC:3.2.1.8 | 1,4-β-xylanase | 10 |  |  |  |
| EC:3.2.1.37 | β-xylosidase | 43 |  |  |  |
| ***Starch*** |  |  |  |  |  |
| EC:3.2.1.1 | α-amylase | 13 |  |  |  |
| EC:3.2.1.52 | β-hexosaminidase | 20 |  |  |  |
| EC:2.4.1.1 | glycogen or starch phosphorylase | | |  | 35 |
| ***Esterase*** |  |  |  |  |  |
| EC:3.1.1.72 | acetyl xylan esterase | |  | 1 |  |
| EC 2.4.1.56 | N-acetylglucosaminyltransferase | | |  | 4 |
